# Volumetric, acoustic, and computational investigation of l-threonine and glycyl-l-threonine interactions in aqueous 1-octyl-3-methylimidazolium bromide solutions

**DOI:** 10.1039/d6ra01144f

**Published:** 2026-06-02

**Authors:** Ravinder Sharma, Sandeep Kumar, Marc Mulamba Tshibangu, Indra Bahadur

**Affiliations:** a Department of Chemistry, National Institute of Technology Hamirpur 177005 India; b Department of Chemical Engineering, Mangosuthu University of Technology Jacobs Durban 4026 South Africa tshibangu.marc@mut.ac.za; c Department of Chemistry, North-West University (Mafikeng Campus) Private Bag X2046 Mmabatho 2735 South Africa bahadur.indra@nwu.ac.za

## Abstract

The present study explores the thermodynamic and molecular interaction behaviour of l-threonine and its dipeptide glycyl-l-threonine in aqueous solutions of the ionic liquid 1-octyl-3-methylimidazolium bromide [OMIm][Br] across a temperature range of 288.15–318.15 K. Accurate measurements of solution density and ultrasonic velocity were undertaken to evaluate key thermodynamic parameters, including apparent molar volume, limiting partial molar properties, transfer volumes, and apparent molar isentropic compressibility. These parameters offer quantitative insights into solute–solvent and solute–solute interactions, highlighting the structure-making or structure-breaking tendencies of the solutes in the ionic liquid–water medium. Results show that both solutes act as structure-makers, with glycyl-l-threonine demonstrating stronger solute–solvent interactions than l-threonine, likely due to the presence of the peptide linkage and increased hydrogen-bonding ability. The effects of temperature and ionic liquid concentration further support the kosmotropic nature of [OMIm][Br], which stabilises hydrophilic and polar groups through hydrogen bonds and electrostatic interactions. To complement the experimental data, molecular docking, molecular dynamics simulations, and DFT-based quantum-chemical analyses were performed to reveal the electronic properties, interaction energies, and hydrogen-bonding patterns that control peptide–ionic liquid interactions. The combined experimental and computational results offer a clear understanding of the solvation dynamics and stabilisation mechanisms of peptides in ionic liquid–water systems. These findings enhance the fundamental understanding of biomolecular behaviour in engineered solvent environments and provide practical insights for developing IL-based platforms for peptide and protein drug-delivery formulations.

## Abstract 2

The study investigates the thermodynamic properties and molecular interactions of l-threonine and glycyl-l-threonine in aqueous 1-octyl-3-methylimidazolium bromide [OMIm][Br] solutions from 288.15 to 318.15 K.

## Graphical abstract

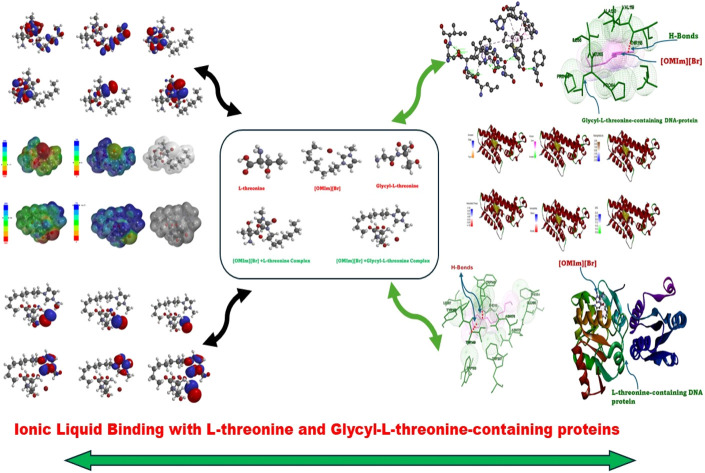

## 1. Introduction

The distinctive physicochemical properties of ionic liquids (ILs), including their minimal vapour pressure, non-flammability, wide electrochemical windows, and high thermal stability, make them excellent alternatives to traditional volatile organic solvents. This set of characteristics, which can be precisely tailored by selecting cations and anions, has driven their use across various technological fields, including catalytic synthesis, separation techniques, sensor development, and electrochemical applications.^1–5^ Beyond these areas, the biocompatibility and solvent-engineering properties of ILs hold transformative potential for biomedical applications, especially for overcoming formulation challenges in delivering biologic agents within the body. Specifically, ILs can serve as multifunctional excipients: their strong kosmotropicity can maintain the tertiary structure of biologics, while their ability to form permeable ionic layers can enhance mucosal penetration. Additionally, their solvation ability allows for the development of in situ-forming gels or polymeric ionic liquid (PIL) hybrids for sustained release, further enhancing the pharmacokinetic profiles and therapeutic effectiveness of biologic drugs.^6–10^ Alongside these biological advances, ionic liquids (ILs) have become a revolutionary class of solvents that have transformed solution chemistry. Characterized by their near-zero vapour pressure, thermal stability, adjustable polarity, and reusability, ILs provide a sustainable and highly adaptable medium for exploring biomolecular processes.^11–17^ Structurally, they are made up of bulky organic cations like imidazolium, ammonium, or pyridinium paired with various anions, from simple halides to more complex groups. This modular design allows ILs to be customized for specific uses by precisely tuning their hydrophilic, hydrophobic, or ionic properties. While their applications have already expanded into fields such as catalysis, separation science, biotechnology, and green chemistry, their role in influencing the thermodynamic behaviour of biomolecules is only beginning to be unraveled.^18–20^

The complex balance of forces that determines the structure and stability of globular proteins remains one of the most intriguing puzzles in molecular biophysics. Proteins exist in a carefully maintained equilibrium, in which an intricate interplay of noncovalent forces, including hydrogen bonds, electrostatic interactions, van der Waals forces, and hydrophobic effects, preserves their native shapes. Collectively, these interactions influence folding pathways, stability limits, and ultimately the biological function of proteins.^21–26^ Even minor disruptions in this balance can cause partial unfolding, misfolding, or complete denaturation—events directly associated with diseases such as Alzheimer's, Parkinson's, and other proteinopathies. As a result, studying these molecular interactions has become a fundamental part of modern chemical and biological research, as it provides a deeper understanding of the basic mechanisms behind protein behaviour.^27,28^ To this end, amino acids and small peptides are often used as simplified molecular models. Their study offers invaluable insights into hydration, solute–solvent and solute–solute interactions, and conformational preferences, serving as a window into the thermodynamic principles governing larger systems of biomacromolecules.^29^

The interactions of ionic liquids with biological macromolecules extend well beyond protein stabilisation. In recent years, considerable attention has been directed toward understanding how ILs interact with DNA and, more importantly, how they influence the delicate interplay between DNA and proteins. Imidazolium-based ILs are known to associate with DNA through multiple pathways.^30,31^ The positively charged imidazolium head group is naturally drawn to the anionic phosphate backbone, while the aromatic ring system can slip between adjacent base pairs through intercalation, and the hydrophobic alkyl chain engages in groove binding. What makes these interactions especially significant is their sensitivity to structural variables, such as alkyl chain length, anion choice, and bulk concentration, meaning that even subtle changes in IL architecture can dramatically alter the nature and strength of DNA binding.^32^ At the level of DNA–protein assemblies, ILs have been shown to tip the balance between stabilisation and disruption of nucleoprotein complexes, with consequences for processes as fundamental as transcription factor recognition and enzymatic catalysis.^33,34^ In this context, amino acids and short peptides bearing hydroxyl, amino, and carboxylate groups, precisely the functionalities present in l-threonine and glycyl-l-threonine occupy the very interaction sites that define protein–DNA recognition. Probing their thermodynamic behaviour in aqueous [OMIm][Br] solutions, therefore, goes beyond isolated solvation studies; it offers a window into how imidazolium-based ILs reshape the molecular environment around peptide fragments that are biologically relevant to DNA-associated processes, with practical implications for the design of peptide–nucleic acid conjugate therapeutics and IL-assisted gene delivery platforms.

Volumetric and acoustic property studies of amino acids and peptides in IL–aqueous mixtures have thus become effective methods for examining the subtle yet essential molecular interactions that govern solvation, hydration, and the organisation of structure.^35–45^ Our current research focuses on l-threonine, an essential, polar amino acid, and its dipeptide glycyl-l-threonine, which is selected for its structural uniqueness and biomedical importance. l-Threonine, characterised by its hydroxyl group in the side chain and high hydrophilicity, plays a crucial role in protein synthesis, collagen and elastin production, immune system regulation, and liver lipid metabolism. Its side chain enables extensive hydrogen bonding and charge–dipole interactions, making it an excellent probe for studying solvation and hydration dynamics. Additionally, l-threonine has direct pharmaceutical significance, being used in parenteral nutrition, in treatments for metabolic and neurodegenerative diseases, and as a starting material for the design of amino acid–drug conjugates that improve bioavailability and reduce toxicity. Conversely, its dipeptide form, glycyl-l-threonine, adds the complexity of a peptide bond, serving as a more realistic model of protein fragments. Beyond its role as a model system, glycyl-l-threonine is utilised in drug delivery systems, peptide-based formulations, and biopharmaceutical manufacturing, where short peptides are valued for their improved solubility, stability, and biological activity. Building on previous studies of imidazolium-based ionic liquids with different alkyl chain lengths ([C_6_mim][Br], [C_8_mim][Br], [C_10_mim][Br], and [C_12_mim][Br]),^46–53^ which showed the strong effect of cationic chain length on volumetric and compressibility properties, this study explores the behaviour of l-threonine and glycyl-l-threonine in aqueous solutions of [C_8_mim][Br].

While previous investigations have examined amino acids in shorter-chain imidazolium ILs such as [C_4_MIm] and [C_6_MIm] derivatives, the specific behaviour of l-threonine and particularly its dipeptide glycyl-l-threonine in [OMIm][Br]–water systems remains largely unexplored. This knowledge gap is significant, as dipeptides are structurally and thermodynamically distinct from free amino acids. The presence of the peptide linkage introduces additional hydrogen-bonding sites, greater conformational flexibility, and a more protein-like character, making it impossible to extrapolate findings from amino acid studies to dipeptide systems. The choice of [OMIm][Br] in this work is not arbitrary. Among the imidazolium-based ILs, the octyl chain (C_8_) represents a critical threshold at which the amphiphilic character of the cation becomes sufficiently pronounced to influence biomolecular hydration shells in a qualitatively different manner compared to shorter-chain analogues. The longer alkyl chain enhances hydrophobic interactions and promotes a more structured organisation around polar biomolecular fragments, making [OMIm][Br] particularly relevant for understanding how amphiphilic ILs stabilise peptide structures, a question with direct implications for IL-based drug delivery system design. Furthermore, while most existing volumetric studies of amino acids in imidazolium ILs remain confined to experimental measurements alone, the present work uniquely addresses this limitation by integrating thermodynamic measurements with molecular docking, molecular dynamics simulations, and DFT-based quantum chemical analysis. This multi-technique approach allows macroscopic thermodynamic observations to be directly connected with molecular-level interaction mechanisms, providing a depth of understanding that experimental measurements alone simply cannot offer.

Recognized for its strong kosmotropic (structure-forming) nature, this IL is a promising candidate for stabilising biomolecules in water. Using accurate measurements of density and ultrasonic velocity over a broad temperature range (288.15–318.15 K), key thermodynamic parameters have been calculated, including apparent molar volume, limiting partial molar properties, transfer volumes, and isentropic compressibility. These parameters serve as sensitive indicators of solute–solvent interactions, revealing whether solutes act as structure makers or breakers in IL–water systems and providing insights into hydration around charged and polar groups. The novelty of this study lies in its combined experimental and computational approach. Along with volumetric and acoustic measurements, *in silico* molecular docking, molecular dynamics simulations, and DFT-based quantum chemical studies have been used to examine electronic properties, hydrogen-bonding patterns, and interaction energies at the molecular level. This integrated methodology supports experimental findings and offers atomistic insights into how dipeptides are solvated and stabilised in IL-water mixtures, a topic that has received little attention to date. Additionally, unlike traditional studies that focus solely on free amino acids, the emphasis on dipeptides provides a more protein-like model, bridging the gap between individual amino acids and larger biomolecules. From an application perspective, the results of this study are highly relevant to pharmaceutical and drug development fields. Understanding how ILs affect amino acid and peptide stability can lead to the development of tunable solvent systems that improve the solubility and stability of peptide therapeutics. This characteristic has significant implications for the formulation of stable protein and peptide drugs, the development of controlled-release delivery systems, and the design of IL-based platforms with reduced degradation and improved pharmacokinetics. Beyond drug development, this study also provides fundamental insights into protein stabilisation, resistance to denaturation, and the engineering of biomimetic solvents.

## 2. Experimental and computational methods

### 2.1 Chemicals and reagents

Analytical grade l-threonine (≥99%) and glycyl-l-threonine (≥99%) were obtained from Merck (Germany) and used as received without further purification. To synthesize the studied ionic liquid, 1-octyl-3-methylimidazolium bromide ([OMim][Br]), the following high-purity reagents were purchased from Sigma-Aldrich (Germany): acetonitrile (≥99%), hexane (≥99%), 1-methylimidazole (≥99%), and 1-bromooctane (≥99%). Before use, all chemicals were vacuum-dried for 24–48 hours to remove moisture and volatile impurities and then stored in a desiccator over phosphorus pentoxide (P_2_O_5_) until needed. This step was taken to ensure the accuracy of density and acoustic measurements, which are highly sensitive to solvent purity. Using high-quality reagents from Merck and Sigma-Aldrich helped reduce experimental variability. A detailed list of chemicals, their purity grades, and suppliers is provided in Table S1 for clarity and reproducibility of record-keeping.

### 2.2 Sample preparation and instrumental

All aqueous solutions were prepared with ultrapure water obtained by triple distillation and degassed, yielding a specific conductance of less than 1 × 10^−6^ S cm^−1^. This ensured minimal ionic contamination and high reproducibility of the physicochemical measurements. The masses of the solutes were measured using a high-precision microbalance, accurate to ±0.00001 g. For samples with 99% purity, the combined standard uncertainty in molality was estimated to be within ±0.001 mol kg^−1^. The density and speed of sound of the solutions were measured with an Anton Paar DSA 5000 M vibrating-tube densimeter equipped with an integrated ultrasonic cell based on a time-of-flight technique. In this setup, ultrasonic pulses of approximately 3 MHz were transmitted through the sample by one piezoelectric transducer and received by another, allowing accurate determination of the sound velocity as the ratio of the propagation distance to the transit time. The densimeter was calibrated before each measurement using triply distilled, degassed water at 293.15 K, ensuring traceability and accuracy. Due to the extreme sensitivity of density and acoustic parameters to temperature fluctuations, the instrument was coupled with a built-in Peltier thermostat that maintained the temperature within ±1.0 × 10^−3^ K throughout the experiments. The instrument offers measurement precisions of 1 × 10^−3^ kg m^−3^ for density and 1 × 10^−2^ m s^−1^ for sound velocity, with expanded uncertainties of ±0.1 kg m^−3^ and ±0.8 m s^−1^, respectively. As an additional quality check, calibration tests were routinely performed using air and triply distilled, degassed water at 293.15 K, confirming the reliability and accuracy of the measurement setup.

### 2.3 Density functional theory (DFT) calculations

Theoretical studies complement experimental findings by providing a molecular-level understanding of solute–solvent interactions. All quantum-chemical calculations were performed using Spartan 20, version 1.1.4 (Wavefunction, Inc., Irvine, CA). The initial interaction configurations for DFT calculations were not constructed arbitrarily but were systematically derived from the top-ranked molecular docking poses obtained prior to quantum-chemical analysis. Multiple poses capturing distinct interaction modes, including hydrogen bonding through the hydroxyl and carboxyl groups, electrostatic interactions with the bromide anion, and hydrophobic contacts with the octyl chain of [OMIm],^+^ were selected as starting geometries to ensure reasonable conformational coverage of the ionic liquid–biomolecule interaction landscape.

Geometry optimisations were carried out using the ωB97X-D range-separated hybrid functional with the 6-311G(d,p) basis set. The ωB97X-D functional was specifically chosen over conventional hybrid functionals such as B3LYP because it incorporates an empirical dispersion correction intrinsically, making it considerably more appropriate for describing the non-covalent interactions governing ionic liquid–peptide complexes, particularly π–π stacking and hydrogen bonding, which are central to the present system.^54^ The integral equation formalism polarisable continuum model (IEFPCM) with water as the solvent was employed to account for bulk solvent effects and better replicate the experimental aqueous environment. Optimised geometries were verified through frequency calculations to confirm the absence of imaginary vibrational modes, ensuring that the reported structures correspond to true energy minima. Following geometry optimisation, frontier molecular orbital (FMO) analysis was performed by calculating the energies of the highest occupied molecular orbital (HOMO) and lowest unoccupied molecular orbital (LUMO). Binding energies for all complexes were calculated as the difference between the total energy of the optimised complex and the sum of the total energies of the individually optimised monomers at the same level of theory, according to: Δ*E* = *E* (complex) – [*E* (monomer A) + *E* (monomer B)], with values reported in kcal mol^−1^.

### 2.4 Molecular docking

To complement the experimental and theoretical studies, *in silico* molecular docking was performed to understand the binding interactions between the ligand and the chosen biomolecular target. The docking process was performed on a Windows system, where the ligand structures of l-threonine, glycyl-l-threonine, and [OMIm][Br] were initially created in ChemSketch and converted to three-dimensional structures. The ligand files were optimised and prepared in AutoDock Tools (ADT), with torsional flexibility and hydrogen atoms added to generate the PDBQT format, and Gasteiger–Marsili partial charges assigned to all atoms. The DNA structure (PDB IDs: 3AJE and 6HOB) was obtained from the Protein Data Bank and prepared by removing water molecules and unnecessary heteroatoms. AutoDock Vina was employed for all docking calculations, which uses an empirical scoring function incorporating steric, hydrogen bonding, electrostatic, and desolvation interaction terms. A grid box of 40 × 40 × 40 Å with a grid spacing of 0.375 Å was centered on the binding region of interest. An exhaustiveness parameter of 32 was employed to ensure comprehensive conformational sampling, and the top 10 binding poses were retained per system based on binding affinity scores. Convergence was confirmed by clustering poses using an RMSD cutoff of 2.0 Å, with consistent reproduction of the top-ranked binding mode across independent runs confirming reliability. The three-dimensional structure of [OMIm][Br] was geometry-optimised prior to docking, and partial charges were validated against previously reported computational studies on imidazolium-based ionic liquids.^55,56^ Binding energies, inhibition constants (*K*_i_), and hydrogen bond interactions were assessed to identify the most favourable complexes. Visualisation and interaction mapping were performed using Discovery Studio Visualizer (Biovia, Dassault Systems), providing detailed insights into hydrogen bonding, π–π stacking, and hydrophobic contacts. The docking results confirmed the experimental observations and highlighted the potential application of these interactions in drug discovery, particularly in designing novel ligands that can selectively target nucleic acid structures.

## 3. Results and discussion

### 3.1 Volumetric analysis and solvation behaviour of l-threonine and glycyl-l-threonine in aqueous ionic liquid [OMim][Br]

**Fig. 1:**
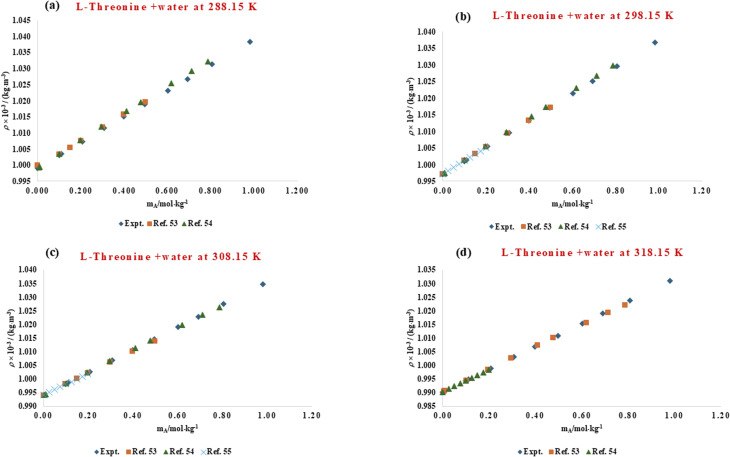
Comparison of experimental and literature^53–55^ values of densities for aqueous solution of l-threonine at different temperatures (288.15 K, 298.15 K, 308.15 K and 318.15 K).

**Fig. 2:**
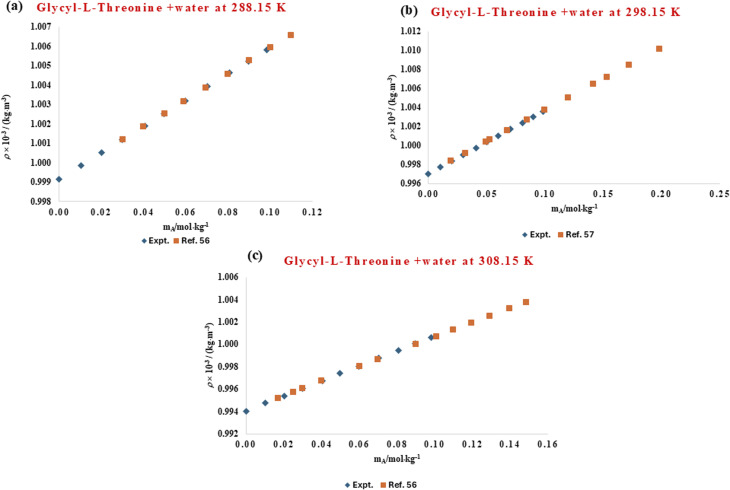
Comparison of experimental and literature^56,57^ values of densities for aqueous solution of glycyl-l-threonine at different temperatures (288.15 K, 298.15 K, and 308.15 K).

The solvation environment of biomolecules is influenced by delicate solute–solvent interactions, which can be effectively studied through volumetric analysis. Hydration phenomena play a crucial role in determining the structural stability, conformational flexibility, and functional behaviour of amino acids and peptides in aqueous environments. The presence of co-solutes, such as ionic liquids, can either enhance or weaken this hydration shell, thereby altering overall solvation dynamics. To understand these molecular interactions, the volumetric behaviour of l-threonine and its dipeptide, glycyl-l-threonine, was systematically examined in aqueous solutions of the imidazolium-based ionic liquid [OMim][Br]. High-precision density measurements were performed across a wide concentration range (0.00–0.40 mol kg^−1^) of [OMim][Br], at four different temperatures (288.15, 298.15, 302.15, and 318.15) K, and at atmospheric pressure (0.1 MPa). The experimentally determined densities shown in Table 2 serve as the basis for deriving thermophysical properties that describe solute–solvent and solute–solute interactions. The experimentally determined densities for the binary-l-threonine-water system exhibit a systematic decrease with increasing temperature. This behaviour is attributed to enhanced molecular thermal motion, which reduces solvent structuring and weakens intermolecular interactions in the aqueous medium. The observed temperature dependence of density is in excellent agreement with previously reported literature data,^53–55^ thereby validating the precision and reliability of the present experimental measurements. A direct graphical comparison between the measured densities and their corresponding literature values, presented in Fig. 1, reveals very close agreement over the investigated temperature range, confirming the consistency of the data. In addition, Fig. 2 demonstrates a strong correlation between the present results and the reported reference values^56,57^ for glycyl-l-threonine solutions, further supporting the accuracy of the experimental methodology and measurement protocol. The measured density data were used to calculate the apparent molar volume (*V*_*ϕ*_), employing the equation previously developed in our research group.1
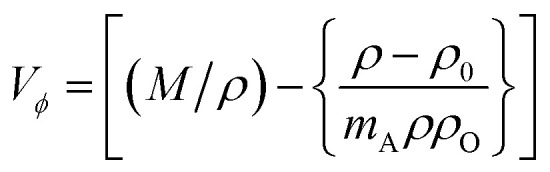


**Fig. 3:**
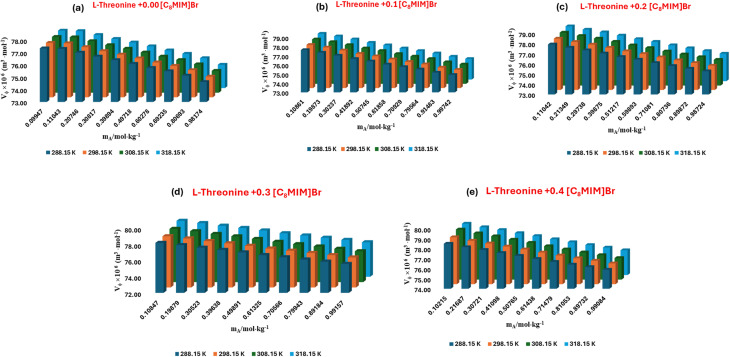
Plots of apparent molar volume (*V*_*ϕ*_) for l-threonine in aqueous 1-octyl-3-methylimidazolium bromide solutions (a) *m*_IL_ = 0.00 mol kg^−1^, (b) *m*_IL_ = 0.10 mol kg^−1^, (c) *m*_IL_ = 0.20 mol kg^−1^, (d) *m*_IL_ = 0.30 mol kg^−1^, (e) *m*_IL_ = 0.40 mol kg^−1^ at different temperatures.

**Fig. 4:**
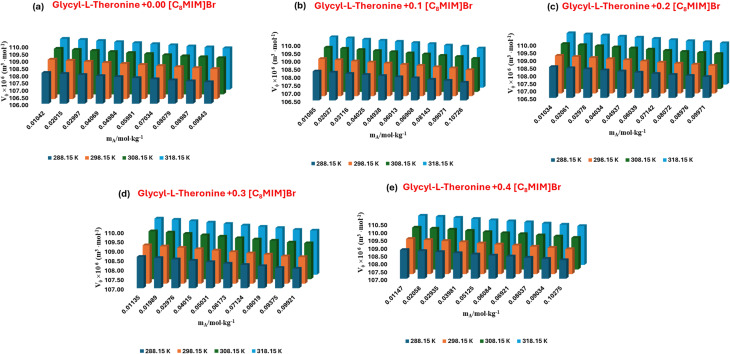
Plots of apparent molar volume (*V*_*ϕ*_) for glycyl-l-threonine in aqueous 1-octyl-3-methylimidazolium bromide solutions (a) *m*_IL_ = 0.00 mol kg^−1^, (b) *m*_IL_ = 0.10 mol kg^−1^, (c) *m*_IL_ = 0.20 mol kg^−1^, (d) *m*_IL_ = 0.30 mol kg^−1^, (e) *m*_IL_ = 0.40 mol kg^−1^ at different temperatures.

The density-derived apparent molar volume (*V*_*ϕ*_) values presented in Table S2 for l-threonine and glycyl-l-threonine in aqueous [OMim][Br] solutions offer a powerful thermodynamic window into solvation dynamics. A consistent increase in (*V*_*ϕ*_), with temperature as shown in Fig. 3 and 4, indicates that elevated thermal energy enhances solvent reorganisation and promotes more extensive solute–solvent interactions. Interestingly, glycyl-l-threonine exhibits larger (*V*_*ϕ*_), values than l-threonine across all studied conditions, underscoring its stronger affinity for the ionic liquid–water environment.

The positive molar volume values observed throughout the system are far from trivial; they represent the net outcome of multiple competing molecular processes.

#### 3.1.1 Microstructural rearrangements

Rather than simply expanding in volume, the positive *V*_*ϕ*_ values indicate a significant restructuring of the local solvent architecture. In the presence of [OMim][Br], the hydration shell around the solute reorganises into a less compact but energetically more favourable arrangement. This reorganisation may involve displacing water molecules from primary hydration layers, redistributing hydrogen bonds, and partially disrupting the tetrahedral water network. Such restructuring results in a measurable increase in effective molar volume, indicating the formation of a new solvent “microenvironment” shaped by the liquid's ionic environment.

#### 3.1.2 Ionic liquid–mediated solvation

The ionic liquid directly influences these volumetric trends. The imidazolium cation can interact electrostatically and through π–cation or hydrogen-bonding interactions with polar groups of amino acids, while its alkyl chain offers a hydrophobic domain capable of associating with nonpolar fragments of the solutes. These dual modes of interaction not only stabilise the solutes but also promote solvent displacement, leading to volumetric expansion. The consistently positive *V*_*ϕ*_ values thus serve as a thermodynamic signature of solvation modulation by [OMim][Br], where both electrostriction and hydrophobic association work together to reshape the local environment.

#### 3.1.3 Balance of solute–solvent interactions

The contrast between l-threonine and glycyl-l-threonine underscores the importance of molecular size and functional group complementarity in solvation. The dipeptide glycyl-l-threonine, with more sites for hydrogen bonding and hydrophobic interactions, interacts more effectively with the ionic liquid–water mixture, resulting in higher *V*_*ϕ*_ values. This finding indicates that the apparent molar volume is highly responsive to small differences in molecular structure, providing a quantitative measure of interaction strength in these ternary systems.

### 3.2 Partial molar volume for l-threonine and glycyl-l-threonine in aqueous ionic liquid [OMim][Br]

The standard partial molar volume is a key thermodynamic parameter that measures the innate volumetric contribution of a solute at infinite dilution. Unlike apparent molar volume, which reflects both solute–solute and solute–solvent interactions, it isolates the pure effect of solute–solvent interactions by removing contributions from solute–solute association. Therefore, it serves as a sensitive indicator of the molecular environment of solutes in complex media, such as amino acids in aqueous ionic liquid solutions. In this study, the values of l-threonine and glycyl-l-threonine were determined in aqueous [OMim][Br] to explore how the ionic liquid influences hydration structure and solvation dynamics. The assessment was performed using least-squares regression analysis on the experimental apparent molar volume data. This method involves extrapolating the *V*^0^_*ϕ*_ values to infinite dilution, based on the concentration dependence described in eqn (2). At this limit, intermolecular crowding is negligible, and the measured *V*^0^_*ϕ*_ becomes a direct thermodynamic fingerprint of solute–solvent interactions and the measured *V*^0^_*ϕ*_ becomes a direct thermodynamic fingerprint of solute–solvent interactions.2
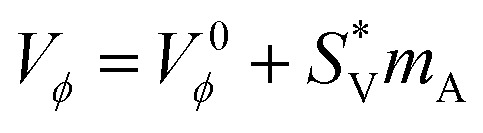


**Fig. 5:**
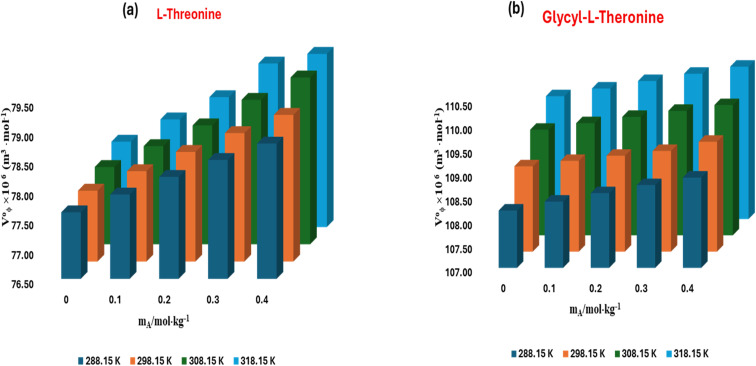
Plots of partial molar volume (*V*^0^_*ϕ*_) for (a) l-threonine (b) glycyl-l-threonine in aqueous 1-octyl-3-methylimidazolium bromide solutions at different temperatures.

The conventional meaning and usage of all symbols employed in this analysis are detailed in our previous work.^50^ The standard partial molar volumes *V*^0^_*ϕ*_ of l-threonine and glycyl-l-threonine in aqueous [OMim][Br] solutions are summarized in Table S3 and illustrated graphically in Fig. 5. The reported values include standard errors, obtained through least-squares regression of the apparent molar volume data. The consistently positive *V*^0^_*ϕ*_ values for both solutes across the studied conditions indicate pronounced solute–ionic liquid interactions within the ternary (solute + ionic liquid + water) system. These interactions become more prominent with increasing temperature, reflecting the cooperative contributions of hydrogen bonding, electrostatic stabilisation, and the unique solvation environment induced by [OMim][Br]. The larger *V*^0^_*ϕ*_ values observed for glycyl-l-threonine relative to l-threonine suggests a more extensive solvation effect, likely attributable to the dipeptide's greater molecular size, additional hydrogen-bonding sites, and enhanced ability to interact with both the cationic and anionic components of the ionic liquid. The temperature dependence of *V*^0^_*ϕ*_ provides further insights into the dynamic nature of solute–solvent interactions. Rising temperatures increase molecular mobility and may facilitate more favourable spatial arrangements of solute and ionic liquid species. This structural reorganisation reduces steric constraints, thereby enhancing the degree of ion–hydrophilic interactions and contributing to the observed increase in *V*^0^_*ϕ*_. From the perspective of the co-sphere overlap model,^58^ changes in solution volume reflect the balance between different types of molecular overlap. Volume expansion is associated with overlaps involving ion-hydrophilic groups, whereas contraction is typically expected for overlaps involving hydrophobic–hydrophobic or ion-hydrophobic groups. The predominance of positive *V*^0^_*ϕ*_ values in the present system clearly demonstrates that ion-hydrophilic interactions dominate, overshadowing contributions from hydrophobic–hydrophobic or ion-hydrophobic associations.

#### 3.2.1 Hydration and solvent reorganization

Positive or elevated values of *V*^0^_*ϕ*_ indicates the disruption of the structured water network around the solute, accompanied by reorganization of hydrogen bonds and redistribution of hydration shells. In systems containing ionic liquids, this effect is amplified, as the bulky imidazolium cation and its hydrophobic alkyl chains perturb the local solvent environment more strongly than conventional electrolytes.

#### 3.2.2 Role of the ionic liquid

The ionic liquid [OMim][Br] contributes dual modes of solvation: electrostatic stabilisation *via* the imidazolium cation–hydroxyl/amine interactions, and hydrophobic association between the alkyl chain of [OMim]+ and the nonpolar segments of the amino acids. These effects manifest as distinct shifts in (*V*^0^_*ϕ*_), providing a thermodynamic signature of how the ionic liquid reshapes solvation compared to pure aqueous systems.

#### 3.2.3 Molecular specificity of solutes

A comparison between l-threonine and glycyl-l-threonine highlights how subtle differences in molecular architecture influence (*V*^0^_*ϕ*_). The dipeptide glycyl-l-threonine, with additional hydrogen-bonding sites and extended hydrophobic surface area, engages in stronger solute–solvent interactions, which is reflected in its higher (*V*^0^_*ϕ*_) values relative to the single amino acid. This underscores the sensitivity of (*V*^0^_*ϕ*_) to molecular size, polarity, and conformational flexibility.

### 3.3 Partial molar volume of transfer volume for l-threonine and glycyl-l-threonine in aqueous ionic liquid [OMim][Br]

The standard partial molar volume of transfer (Δ*V*^0^_*ϕ*_) is a key thermodynamic parameter that quantifies the volumetric consequences of transferring a solute from one solvent environment into another. In the present study, (Δ*V*^0^_*ϕ*_) was determined for l-threonine and glycyl-l-threonine to probe the effect of introducing the ionic liquid [OMim][Br] into the aqueous medium. Specifically, it was calculated as the difference between the standard partial molar volume of the solute in aqueous [OMim][Br] solution and that in pure water. This approach allows a rigorous evaluation of the influence of [OMim][Br] on solute solvation, highlighting the role of ionic-liquid-induced structural perturbations in modulating the system's volumetric properties. The partial molar volume of transfer can be expressed as

3 Δ*V*^0^_*ϕ*_ = *V*^0^_*ϕ*_ (in aqueous[OMIm][Br]solution) − *V*^0^_*ϕ*_(in water)

**Fig. 6:**
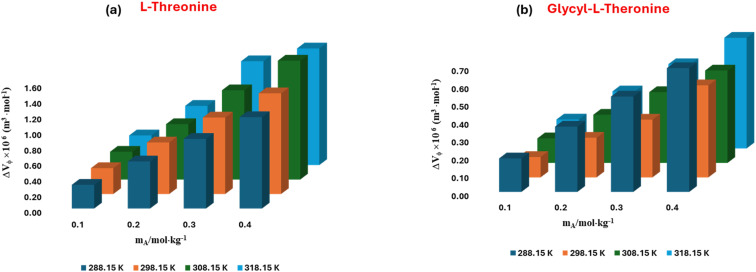
Plots of partial molar volume of transfer (*V*^0^_*ϕ*_) for (a) l-threonine (b) glycyl-l-threonine in aqueous 1-octyl-3-methylimidazolium bromide solutions, at different temperatures.

Eqn (3) not only elucidates the interactions between solute and co-solvent species, but it also provides a robust thermodynamic framework for isolating the influence of the co-solvent from solute–solute contributions. The standard partial molar volumes of transfer (Δ*V*^0^_*ϕ*_) for l-threonine and glycyl-l-threonine in aqueous [OMim][Br] solutions were calculated, and the results are summarized in Table S4 and illustrated in Fig. 6. The transfer volumes were consistently positive across the investigated conditions, highlighting the favourable nature of solute–co-solvent interactions in these systems. The positive (Δ*V*^0^_*ϕ*_) values suggest that the polar functional groups of both the ionic liquid [OMim][Br] and the amino acid/dipeptide engage in cooperative interactions, primarily through hydrogen bonding and electrostatic forces. Such interactions enhance the solvation environment of the solute, indicating that both l-threonine and glycyl-l-threonine behave as structure-promoting (structure-making) agents in the mixed solvent system. This observation is consistent with the known ability of ionic liquids to modulate the local water structure and facilitate more extensive solute–solvent coupling.

The co-sphere overlap model further rationalizes these findings. According to this model, positive transfer volumes arise when ion–hydrophilic interactions dominate, leading to an effective expansion of the solvation sphere and an increase in system volume. In the present study, the observed positive (Δ*V*^0^_*ϕ*_) values confirm that favourable overlaps between the hydration spheres of the solute and the ionic liquid prevail over hydrophobic–hydrophobic or ion–hydrophobic interactions. This highlights the role of [OMim][Br] in reinforcing solute hydration and in contributing to the system's enhanced volumetric behavior. The intermolecular interactions between l-threonine/glycyl-l-threonine and the ionic liquid [OMim][Br] can be systematically interpreted using the co-sphere overlap model, which categorises solute–solvent and solvent–solvent interactions into three principal types.

#### 3.3.1 Hydrophilic–hydrophilic interactions

These interactions occur between the charged centers of [OMim][Br] (imidazolium cation and bromide anion) and the polar/charged functional groups of the solutes. Specifically, the protonated amino group (–NH_3_^+^), the deprotonated carboxylate group (–COO^−^), and the peptide linkage (–CONH) of glycyl-l-threonine can establish strong ion–dipole and hydrogen-bonding interactions with the ionic liquid. Such cooperative interactions strengthen the hydration shell of the solute and lead to an increase in the partial molar volume of transfer (Δ*V*^0^_*ϕ*_) since the overlap of hydration co-spheres associated with polar groups promotes structural expansion of the solvent environment.

#### 3.3.2 Hydrophilic–hydrophobic interactions

These involve cross-interactions between polar groups of the solutes (–NH_3_^+^, –COO^−^, or –CONH) and the hydrophobic moieties of [OMim][Br] (notably, the octyl chain of the imidazolium cation), or alternatively between the hydrophobic side chains of the biomolecules and polar regions of the ionic liquid. Such mixed interactions often disrupt the local hydrogen-bonding network of water, thereby producing a negative contribution to (Δ*V*^0^_*ϕ*_) as the structural organization of the hydration layer becomes less efficient.

#### 3.3.3 Hydrophobic–hydrophobic interactions

These interactions occur between the non-polar alkyl chains of [OMim][Br] and the hydrophobic groups of the biomolecules. The overlap of hydrophobic hydration co-spheres generally results in a volume contraction (negative (Δ*V*^0^_*ϕ*_)), since the exclusion of water molecules from hydrophobic domains reduces overall structural order, leading to system compaction.

According to the co-sphere overlap model, the sign and magnitude of (Δ*V*^0^_*ϕ*_) reflect the balance of these interactions. Positive (Δ*V*^0^_*ϕ*_) values, as observed in this study, are characteristic of systems where hydrophilic–hydrophilic interactions dominate over both hydrophilic–hydrophobic and hydrophobic–hydrophobic contributions. This finding indicates that the strong ion–dipole and hydrogen-bonding interactions between the solutes (l-threonine and glycyl-l-threonine) and [OMim][Br] outweigh any disruptive effects arising from hydrophobic contacts. The predominance of hydrophilic–hydrophilic interactions suggests that both amino acid and dipeptide molecules act as structure-promoting (structure-making) agents in the ternary solvent system. The ionic liquid plays a crucial role in stabilizing these interactions by providing a complementary solvation environment, wherein its polar head groups reinforce solute hydration while its hydrophobic moieties modulate local solvent structuring. Consequently, the observed positive transfer volumes highlight the synergistic solvation dynamics that govern the molecular organization of the ternary system.

### 3.4 Temperature-driven changes in partial molar volume

To systematically examine the influence of temperature on the apparent molar volumes at infinite dilution (*V*^0^_*ϕ*_), a generalised polynomial fitting approach was employed. This mathematical framework not only facilitated the accurate representation of the experimental data but also enabled the extraction of quantitative information regarding the thermal response of solute–solvent interactions. By analysing the variation of (*V*^0^_*ϕ*_) with temperature, it was possible to identify distinct trends and subtle deviations, thereby providing deeper insights into the underlying molecular interactions, solvation dynamics, and structural reorganisations occurring within the ternary system. Such an approach is particularly valuable, as it allows the correlation of volumetric properties with thermodynamic behaviour, highlighting the temperature-dependent balance between hydrophilic, hydrophobic, and ion–solute interactions. The general polynomial equation used for this analysis is given by:

4 *V*^0^_*ϕ*_ = *a* + *b*(*T* − *T*_ref_) + *c*(*T* − *T*_ref_)^2^

where, *T* denotes the absolute temperature, while *a*, *b*, and *c* represent the regression coefficients obtained through least-squares analysis.

The use of this polynomial representation offers a versatile and robust means of describing the temperature dependence of (*V*^0^_*ϕ*_). This approach not only ensures a statistically rigorous fit to the experimental data but also enhances the interpretability of thermodynamic trends, providing valuable insights into the molecular–scale interactions and structural reorganizations that govern solute–solvent behavior in the studied system. The polynomial analysis of the experimental data revealed clear trends in the temperature dependence of the standard partial molar volumes (*V*^0^_*ϕ*_), offering valuable insights into the thermal response of the solute molecules and their interaction dynamics in the solution environment. The empirical coefficients (*a*, *b*, *c*) obtained for l-threonine and glycyl-l-threonine in aqueous 1-octyl-3-methylimidazolium bromide are summarized in Table 5. These constants provide a quantitative framework for describing how solute–solvent interactions evolve with increasing temperature. Notably, the positive values of *a*, *b*, and *c* for both solutes indicate that (*V*^0^_*ϕ*_) increases systematically with temperature, highlighting the significant role of thermal energy in modulating hydration structures and intermolecular associations within the ionic liquid–water medium. The positive values of these coefficients further suggest that solute molecules experience enhanced volumetric expansion with increasing temperature, a behaviour linked to the progressive disruption of hydrogen-bonded networks and the reorganisation of the solvation shell. Such temperature-driven effects are particularly important for understanding the physicochemical behaviour of amino acids and small peptides in ionic liquid systems, where subtle balances between hydrophilic and hydrophobic interactions dictate solution properties.

To assess the reliability of the polynomial model, theoretical values of (*V*^0^_*ϕ*_) were calculated using the derived constants, and the corresponding absolute standard deviations (*σ*) were evaluated. The (*σ*) values, also listed in Table 5, demonstrate excellent agreement between the experimental and calculated data, thereby confirming the robustness and predictive capability of the polynomial approach for describing temperature-dependent volumetric properties in these complex systems.5
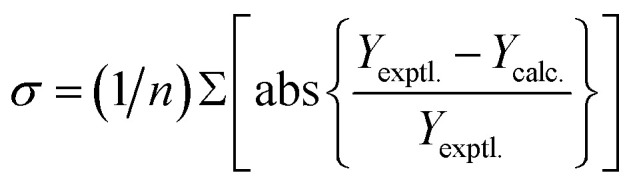


Table 1 clearly shows that the polynomial formalism provides a highly reliable framework for predicting the volumetric behaviour of ionic liquids, amino acids, and dipeptides, even when experimental variations are relatively small. The robustness of this approach lies in its ability to capture subtle changes in volumetric properties with temperature, thereby offering a quantitative description of solute–solvent interactions under thermal perturbation. The temperature dependence (*T*) of the standard partial molar volume (*V*^0^_*ϕ*_) is best represented as a function of the absolute temperature, ensuring thermodynamic consistency and enabling precise modelling of molecular-level behaviour. By employing this representation, the polynomial equation not only characterizes the continuous evolution of (*V*^0^_*ϕ*_) with increasing temperature but also accounts for the non-linear contributions arising from complex solvation phenomena, such as the disruption of hydrogen-bonding networks and the reorganisation of hydration shells. This methodology therefore provides deeper insight into the thermal sensitivity of amino acids and dipeptides in ionic liquid–aqueous systems, highlighting how even small structural differences in the solute can give rise to distinct volumetric responses. Consequently, the use of absolute temperature in this analysis provides a rigorous, predictive description of temperature-dependent solute–solvent interactions, thereby strengthening the reliability of volumetric studies in complex fluid systems.

**Table 1:** Analysis of empirical properties of l-threonine and glycyl-l-threonine in aqueous media of 1-octyl-3-methylimidazolium bromide

a *m*_B_/(mol kg^−1^)	*a* ×10^6^/(m^3^ mol^−1^)	*b* × 10^6^/(m^3^ mol^−1^ K^−1^)	*c* × 10^6^/(m^3^ mol^−1^ K^−2^)	ARD (*σ*)
**l** **-Threonine**				
0.000	77.70	0.00915	0.00016	0.00356
0.100	78.03	0.01177	0.00015	0.00575
0.200	78.36	0.01451	0.00013	0.00215
0.300	78.69	0.02148	0.00040	0.08176
0.400	79.05	0.02492	0.00022	0.00373

**Glycyl-** **l** **-threonine**				
0.000	108.78	0.05113	0.00056	0.00147
0.100	108.90	0.04816	0.00032	0.00246
0.200	109.03	0.04541	0.00008	0.00385
0.300	109.14	0.04287	0.00015	0.08472
0.400	109.30	0.04198	0.00014	0.00583

a *m*_B_ is the molality of aqueous solutions of 1-octyl-3-methylimidazolium bromide.

### 3.5 Analysis of structure-forming and structure-breaking abilities

To gain deeper insight into the temperature dependence of the volumetric properties of the studied systems, the limiting apparent molar expansibility (*E*^0^_*ϕ*_) was evaluated. This parameter, which quantifies the change in apparent molar volume per unit change in temperature, serves as a sensitive probe for characterising solute–solvent interactions under thermal perturbations. The calculation of (*E*^0^_*ϕ*_) was carried out using eqn (6), which relates the expansibility directly to the first derivative of the apparent molar volume with respect to absolute temperature.

6 *E*^0^_*ϕ*_ = (∂*V*^0^_*ϕ*_/∂*T*)*p* = *b* + 2*c*(*T* − *T*_ref_)

The expansibility parameters provide critical information about the structural reorganisations within the solvation shell as temperature varies. A positive reflects enhanced molecular expansion and is typically associated with the disruption of solvent–solute hydrogen-bond networks or increased mobility of solvent molecules in the hydration shell. Conversely, negative values indicate contraction or stronger ordering effects within the local environment of the solute. By systematically evaluating these parameters, it is possible to distinguish between structure-promoting and structure-breaking contributions from the ionic liquid and biomolecules, thereby providing a more comprehensive thermodynamic characterisation of the system. By employing these thermodynamic concepts, it becomes possible to elucidate the subtle intermolecular forces governing solute–solvent interactions and to develop a comprehensive understanding of the solution's structural and dynamic behaviour across varying thermal conditions.7
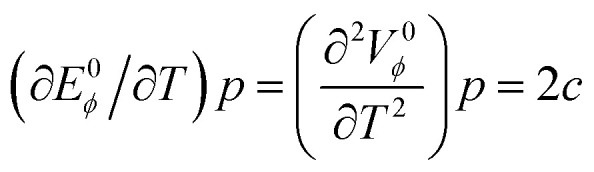


Table 2 reports the positive values of the limiting partial molar expansibility, (∂*E*^0^_*ϕ*_/∂*T*)*p* for both dipeptides and amino acids in the investigated system, thereby underscoring their potential role in promoting structural organisation within the solvent medium. The positive expansibility values suggest that these biomolecules exhibit a tendency to form structure, consistent with their ability to enhance solvent ordering through favourable solute–solvent interactions. Notably, the observed variations in these parameters with increasing concentrations of the ionic liquid [OMim][Br] highlight the complex interplay of packing or “caging” effects, which significantly modulate the spatial arrangement and dynamic behaviour of solvent molecules around the solutes. The interpretation of these results is further strengthened by employing Hepler's criterion, which provides a thermodynamic framework to distinguish between structure-making and structure-breaking behavior of solutes in aqueous and mixed solvent systems. According to this framework, the positive slopes of (∂*E*^0^_*ϕ*_/∂*T*)*p* affirm that both l-threonine and glycyl-l-threonine act as structure-makers, enhancing the degree of organisation in the solvent network. By examining the limiting partial molar expansibility within this context, we gain deeper insights into the molecular mechanisms by which solutes influence solvent structure either by reinforcing hydrogen-bonding interactions and cooperative hydration (structure-making) or by disrupting the solvent network (structure-breaking). Thus, the present findings provide compelling evidence that the solutes studied predominantly stabilise the solvent structure, reflecting the dominance of hydrophilic–hydrophilic interactions in the ternary system.

**Table 2:** Temperature-dependent limiting apparent molar expansibility (*ϕ*^0^_E_) values of l-threonine and glycyl-l-threonine in aqueous 1-octyl-3-methylimidazolium bromide

a *m*_B_/(mol kg^−1^)	*ϕ* ^0^_E_ × 10^6^/(m^3^ mol^−1^ K^−1^)				(∂*ϕ*^0^_E_/∂*T*)*p*/(m^3^ mol^−1^ K^−2^)
	*T* = 288.15 K	*T* = 298.15 K	*T* = 308.15 K	*T* = 318.15 K	
**l** **-Threonine**					
0.000	0.00602	0.00915	0.01227	0.01539	0.00031
0.100	0.00885	0.01177	0.01468	0.01760	0.00029
0.200	0.01182	0.01451	0.01721	0.01990	0.00027
0.300	0.01345	0.02148	0.02950	0.03753	0.00080
0.400	0.02059	0.02492	0.02926	0.03360	0.00043

**Glycyl-** **l** **-threonine**					
0.000	0.06235	0.05113	0.03991	0.02869	0.00112
0.100	0.05450	0.04816	0.04183	0.03549	0.00063
0.200	0.04699	0.04541	0.04384	0.04227	0.00016
0.300	0.03980	0.04287	0.04594	0.04901	0.00031
0.400	0.03923	0.04198	0.04473	0.04748	0.00028

a *m*_B_ is the molality of aqueous solutions of 1-octyl-3-methylimidazolium bromide.

## 4. Compressibility properties of l-threonine/glycyl-l-threonine

### 4.1 Molecular interaction analysis through experimental acoustic velocity and derived isentropic compressibility

The ultrasonic velocity (*u*) of l-threonine and glycyl-l-threonine was systematically determined in aqueous solutions of the ionic liquid 1-octyl-3-methylimidazolium bromide ([OMim][Br]) over a wide temperature range (288.15, 298.15, 308.15, and 318.15 K) at a constant pressure of 0.1 MPa. The experimentally measured values of *u* are summarised in Table S5. For the interpretation of these acoustic data, the Newton–Laplace relation was employed, which mathematically correlates the velocity of sound (*u*) with the density (*ρ*) of the solution and its isentropic compressibility (*K*_s_). This relation is expressed as:

8 *K*_s_ = 1/(*u*^2^*ρ*)

The apparent molar isentropic compressibility (*K*_*ϕ*,s_) of the solute in a ternary system can be rigorously evaluated using well-established thermodynamic relations that incorporate experimental measurements of solution density and ultrasonic velocity.9
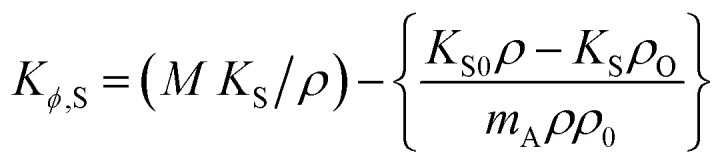


This parameter serves as a highly sensitive probe of solute–solvent interactions, as it quantifies the solute's contribution to the medium's overall compressibility under isentropic (adiabatic, constant entropy) conditions. In essence, (*K*_*ϕ*,s_) reflects the degree to which the presence of a solute perturbs the compressibility of the solvent environment, thereby offering valuable insight into molecular packing effects, hydration/solvation dynamics, and the strength of intermolecular forces.

**Fig. 7:**
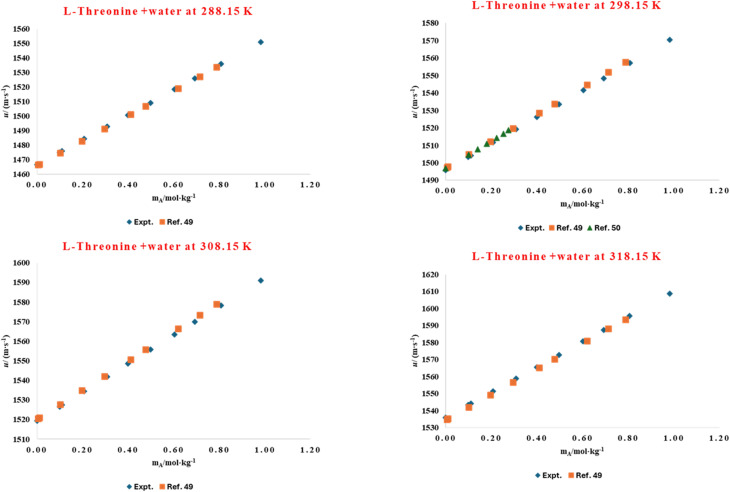
Comparison of experimental and literature^53,54^ values of speed of sound for aqueous solution of l-threonine at different temperatures.

**Fig. 8:**
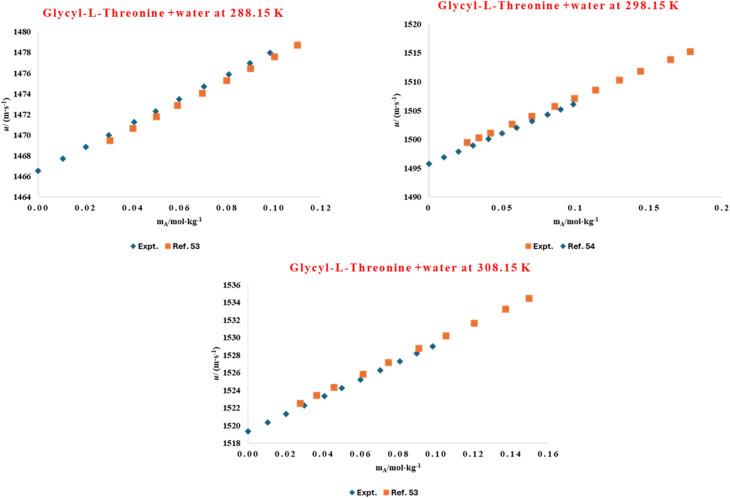
Comparison of experimental and literature^59,60^ values of speed of sound for aqueous solution of glycyl-l-threonine at different temperatures.

**Fig. 9:**
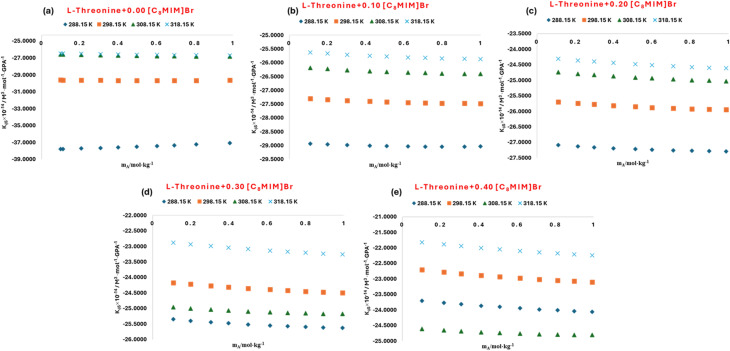
Plots of apparent molar isentropic compressibility (*K*_*ϕ*,s_) for l-threonine in aqueous 1-octyl-3-methylimidazolium bromide solutions (a) *m*_IL_ = 0.00 mol kg^−1^ (b) *m*_IL_ = 0.10 mol kg^−1^ (c) *m*_IL_ = 0.20 mol kg^−1^ (d) *m*_IL_ = 0.30 mol kg^−1^ (e) *m*_IL_ = 0.40 mol kg^−1^ at different temperatures.

**Fig. 10:**
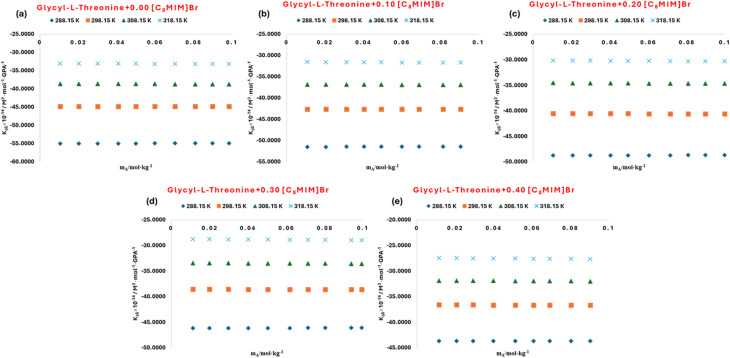
Plots of apparent molar isentropic compressibility (*K*_*ϕ*,s_) for glycyl-l-threonine in aqueous 1-octyl-3-methylimidazolium bromide solutions (a) *m*_IL_ = 0.00 mol kg^−1^ (b) *m*_IL_ = 0.10 mol kg^−1^ (c) *m*_IL_ = 0.20 mol kg^−1^ (d) *m*_IL_ = 0.30 mol kg^−1^ (e) *m*_IL_ = 0.40 mol kg^−1^ at different temperatures.

The ultrasonic velocity (*u*) of l-threonine in aqueous solution was measured across the studied temperature range and graphically presented in Fig. 7, where the data were compared with reported literature values.^53,54^ A similar comparative analysis for aqueous glycyl-l-threonine is illustrated in Fig. 8, benchmarked against previously published datasets.^59,60^ In both cases, the experimental results demonstrate excellent agreement with literature values over a wide concentration range, confirming the reliability of the present measurements and validating the reproducibility of the adopted methodology. The derived values of the apparent molar isentropic compressibility (*K*_*ϕ*,s_) together with the corresponding sound speed (*u*) for l-threonine and glycyl-l-threonine in aqueous [OMim][Br] are summarized in Table S5, and graphically represented in Fig. 9 and 10. A notable feature of the data is the occurrence of consistently negative values of (*K*_*ϕ*,s_) throughout the investigated temperature range. Interestingly, the magnitude of these negative values diminishes progressively with increasing temperature and ionic liquid concentration, suggesting that solute–solvent interactions and packing dynamics are modulated by temperature and composition. The presence of negative (*K*_*ϕ*,s_) values in ternary mixtures of [OMim][Br] + water with l-threonine and glycyl-l-threonine is highly significant, as it reflects a unique compressibility behaviour that deviates from ideal solution properties. These negative values imply that the introduction of solute molecules leads to contraction of the system rather than expansion, a phenomenon attributable to specific intermolecular interactions and molecular-level structural reorganisations. The observed behavior can be rationalised by considering the following interactional contributions.

#### 4.1.1. Ion–dipole interactions

Ionic liquids such as [OMim][Br], composed of a bulky imidazolium cation and bromide anion, generate strong electrostatic fields in aqueous environments. These fields interact strongly with the dipolar water molecules through ion–dipole forces, driving structural reorientation of the hydration shell around the ionic species. The reorganisation of water molecules under the influence of these electrostatic interactions results in efficient packing and reduced free volume, manifesting as a contraction of the overall solution volume. This contraction is directly observed as negative (*K*_*ϕ*,s_). Moreover, the balance between ion–dipole forces and the intrinsic hydrogen-bonding network of water determines the extent of this contraction. At elevated temperatures, increased thermal agitation weakens ion–dipole ordering, leading to a gradual reduction in the magnitude of (*K*_*ϕ*,s_).

#### 4.1.2. Hydrogen bonding and solute–solvent network formation

The incorporation of amino acids (l-threonine) and dipeptides (glycyl-l-threonine) introduces functional groups (–OH, –NH_2_, and –CONH–) capable of engaging in extensive hydrogen-bonding interactions with surrounding water molecules and, in some cases, with the ionic liquid constituents. These hydrogen bonds act as directional, cooperative forces that promote structural ordering within the solution. The resulting three-dimensional hydrogen-bonding network reduces molecular mobility and enhances the system's compactness. Under compression, or with increasing solute concentration, these networks can undergo dynamic restructuring to optimize packing efficiency, thereby amplifying the contraction effect and reinforcing the observed negative compressibility.

Notably, amino acids and peptides often act as structure-making solutes, promoting water structuring through hydrogen-bond stabilisation. In the current system, this structure-making effect synergises with the ion–dipole ordering induced by [OMim][Br], leading to a pronounced reduction in isentropic compressibility. However, with increasing temperature, the disruption of hydrogen-bond networks and enhanced molecular motion result in a gradual attenuation of these effects, consistent with the less negative (*K*_*ϕ*,s_) values at higher temperatures.

#### 4.1.3. Molecular packing and caging effects

Beyond direct interactions, the combined influence of ion–dipole and hydrogen bonding fosters the formation of packing or caging effects, in which water molecules are confined to localised, well-ordered microenvironments around the solutes. These cages contribute to the system's rigidity and compactness, thereby lowering its compressibility. At higher concentrations of [OMim][Br], competition between water structuring around ions and hydrogen bonding with amino acid residues results in a delicate balance, which explains the observed variability and the diminishing magnitude of negative (*K*_*ϕ*,s_) values at elevated concentrations.

### 4.2 Limiting isentropic compression of l-threonine/glycyl-l-threonine

According to eqn (9), the isentropic compressibility factor (*K*^0^_*ϕ*,s_) which serves as a thermodynamic descriptor of the system's departure from ideal behaviour under isentropic conditions, exhibits a pronounced divergence with increasing molal concentration of the solution. This deviation reflects the progressive departure from ideality that arises as solute–solvent and solute–solute interactions intensify at higher concentrations.10
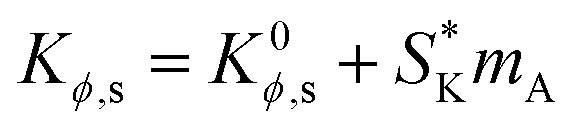


At low molalities, the solution behaviour is primarily dominated by solute–solvent interactions, in which ion–dipole forces and hydrogen bonding stabilise the system and promote partial structural ordering of solvent molecules. However, as the concentration increases, these primary interactions are supplemented and, in some cases, competitively disrupted by solute–solute associations and cooperative rearrangements within the solvent matrix. The result is a complex interplay of forces that manifests in measurable changes in compressibility.

The experimental slope parameters 
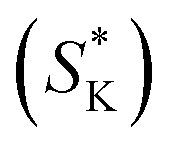
 and (*K*^0^_*ϕ*,s_) together with their associated standard errors, were evaluated using least-squares regression analysis, and the results are presented in Table 3. The negative values of these parameters, particularly for l-threonine and glycyl-l-threonine at lower temperatures, strongly suggest pronounced solute–solvent interactions. These interactions are primarily mediated by hydrogen bonding between amino acid/dipeptide functional groups and the water molecules and are enhanced at lower thermal energies, where solvent structuring is more stable.

**Table 3:** Measurements of the limiting apparent molar isentropic compression, *K*_*ϕ*,s_, and experimental slopes, 
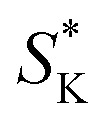
 for l-threonine and glycyl-l-threonine carried out in aqueous solutions of 1-octyl-3-methylimidazolium bromide across temperatures of 288.15 to 318.15 K at an experimental pressure of, *p* = 0.1 MPa

a *m*_B_/(mol kg^−1^)	288.15 K	298.15 K	308.15 K	318.15 K
**l** **-Threonine**				
***K*** ^**0**^_***ϕ*,s**_ **×10^−^** ^**6**^ **/(m** ^**3**^ **mol^−^** ^**1**^ **GPa^−^** ^**1**^ **)**				
0.000	−37.8995(±0.0079)	−29.6277(±0.0103)	−26.5794(±0.0106)	−26.4624(±0.0104)
0.100	−28.9426(±0.0115)	−27.3074(±0.0114)	−26.1867(±0.0113)	−25.6219(±0.0112)
0.200	−27.0925(±0.0110)	−25.6912(±0.0108)	−24.7283(±0.0107)	−24.2933(±0.0107)
0.300	−25.3421(±0.0110)	−24.1529(±0.0108)	−24.9597(±0.0105)	−22.8548(±0.0107)
0.400	−23.6879(±0.0111)	−22.6906(±0.0109)	−24.6147(±0.0104)	−21.7933(±0.0108)

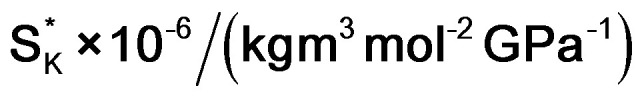				
0.000	0.8249(±0.0145)	−0.0456(±0.0187)	−0.2941(±0.0193)	−0.2643(±0.0190)
0.100	−0.1192(±0.0184)	−0.1944(±0.0182)	−0.2445(±0.0180)	−0.2705(±0.0179)
0.200	−0.2219(±0.0177)	−0.2838(±0.0174)	−0.3254(±0.0173)	−0.3454(±0.0171)
0.300	−0.3157(±0.0177)	−0.3663(±0.0175)	−0.2439(±0.0169)	−0.4309(±0.0177)
0.400	−0.3985(±0.0177)	−0.4395(±0.0175)	−0.2176(±0.0166)	−0.4787(±0.0173)

**Glycyl-** **l** **-threonine**				
***K*** ^**0**^_***ϕ*,s**_ **×10^−^** ^**6**^ **/(m** ^**3**^ **mol^−^** ^**1**^ **GPa^−^** ^**1**^ **)**				
0.000	−55.0303(±0.0003)	−44.7933(±0.0004)	−38.5984(±0.0004)	−33.0062(±0.0004)
0.100	−51.5021(±0.0004)	−42.6202(±0.0004)	−36.8072(±0.0005)	−31.5376(±0.0005)
0.200	−48.7592(±0.0003)	−40.5480(±0.0004)	−34.5498(±0.0004)	−30.1259(±0.0004)
0.300	−46.1586(±0.0003)	−38.5723(±0.0004)	−33.4515(±0.0004)	−28.7695(±0.0004)
0.400	−43.6921(±0.0003)	−36.6079(±0.0004)	−31.8805(±0.0004)	−27.4660(±0.0004)

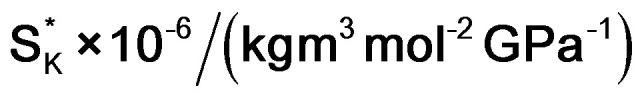				
0.000	1.1719(±0.0060)	−0.3818(±0.0068)	−1.1860(±0.0070)	−1.8929(±0.0072)
0.100	0.7740(±0.0069)	−0.5392(±0.0077)	−1.2913(±0.0079)	−1.9585(±0.0080)
0.200	0.5138(±0.0061)	−0.6933(±0.0067)	−1.4767(±0.0069)	−2.0283(±0.0070)
0.300	0.2803(±0.0060)	−0.8293(±0.0065)	−1.4894(±0.0067)	−2.0862(±0.0068)
0.400	0.0666(±0.0063)	−0.9661(±0.0068)	−1.5731(±0.0069)	−2.1387(±0.0070)

a *m*_B_ is the molality of aqueous solutions of 1-octyl-3-methylimidazolium bromide.

With increasing temperature, the persistence of negative values reflects a gradual attenuation of these attractive forces, likely due to the thermal disruption of hydrogen-bonded networks. As water molecules are progressively released from the solvation shells into the bulk phase, the local ordering surrounding the solute diminishes, reducing the strength of solute–solvent coupling. This weakening of hydrogen bonding and hydration effects accounts for the observed temperature-dependent trends in isentropic compressibility.

The occurrence of negative values of 
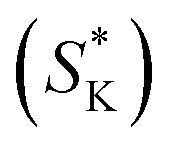
 and (*K*^0^_*ϕ*,s_), in the ternary aqueous mixtures containing [OMim][Br], l-threonine, and glycyl-l-threonine highlights an anomalous compressibility response, indicative of non-ideal molecular interactions and possible structural rearrangements within the solution. Such behaviour deviates from classical expectations and underscores the system's complex thermodynamic nature.^61^

#### 4.2.1 Influence of ionic species

The presence of the ionic liquid [OMim][Br] introduces cations and anions that engage in strong electrostatic interactions with both water and biomolecules. These ionic species alter the local compressibility by modifying the pressure response of the medium *via* ion–dipole interactions, ion pairing, and possible cluster formation. Processes such as transient complexation and dissociation further modulate the system's dynamic equilibrium, producing measurable deviations in compressibility.

#### 4.2.2 Role of water as solvent

Water contributes critically to the observed anomalies due to its extensive hydrogen-bonding network and polarity. The solvent's structural adaptability facilitates the formation of highly organized hydration shells around solutes. Perturbation of this network by ionic liquid species and biomolecules induces cooperative effects, resulting in novel solute–solvent arrangements. These rearrangements alter solvation dynamics, thereby influencing compressibility in ways not typically observed in simpler aqueous systems.

#### 4.2.3 Conformational flexibility of biomolecules

Biomolecules such as glycyl-l-threonine exhibit conformational adaptability, particularly around peptide linkages. Variations in temperature, pressure, or ionic environment can drive structural transitions that alter molecular packing and the compressibility response. Such conformational fluctuations may amplify the sensitivity of compressibility to solute concentration and temperature.

#### 4.2.4 Unusual molecular interactions

Negative compressibility values may also arise from atypical combinations of intermolecular forces, such as ionic interactions, hydrogen bonding, and van der Waals forces, that synergize in non-classical ways. Instead of the expected increase in molecular disorder upon compression, these interactions may enforce local ordering, leading to a paradoxical decrease in entropy. This anomalous response reflects cooperative molecular rearrangements or pressure-induced restructuring of hydrogen-bond networks, offering deep insight into the complex interplay of forces within this multicomponent system.

### 4.3 Compression of transfer and partial molar quantities of l-threonine/glycyl-l-threonine

The term “compression of transfer” (Δ*K*^0^_*ϕ*,s_) is a thermodynamic parameter that reflects the change in partial molar isentropic compressibility of a solute when it moves from one medium to another, typically from pure solvent to a mixed solvent system. Partial molar quantities describe the individual contributions of each component to a mixture's thermodynamic properties, thereby providing molecular-level insight into solute–solvent interactions. In this context, transfer compression quantifies how the local environment of a solute, such as an amino acid or an ionic liquid, modifies its compressibility under isentropic conditions. This parameter is particularly informative for systems containing ionic liquids and amino acids, where solvation is governed by electrostatic interactions, ion–dipole forces, and hydrogen bonding. Variations in the compression of transfer highlight changes in solute hydration, disruption or stabilisation of hydrogen-bonded networks, and possible structural reorganisation within the solution. Thus, analysis of this property not only reveals the strength and nature of solute–solvent interactions but also provides a deeper understanding of molecular packing, solvation dynamics, and structural adaptability in aqueous and mixed solvent systems.

Table 4 summarizes the values of the partial molar isentropic compression of transfer, Δ*K*^0^_*ϕ*,s_ for l-threonine and glycyl-l-threonine in the ternary system composed of [OMim][Br] and water, calculated as follows:

11 Δ*K*^0^_*ϕ*,s_ = (in aqueous[OMIm][Br]) − *K*^0^_*ϕ*,s_ (in water)

**Table 4:** Apparent molar isentropic compression of transfer Δ*K*^0^_*ϕ*,s_ of l-threonine and glycyl-l-threonine measured in 1-octyl-3-methylimidazolium bromide aqueous solutions at four temperatures (288.15, 298.15, 308.15, and 318.15 K) under constant pressure (*p* = 0.1 MPa)

a *m*_B_/(mol kg^−1^)	Δ*K*^0^_*ϕ*,s_ × 10^−6^/(m^3^ mol^−1^ GPa^−1^)			
	*T* = 288.15 K	*T* = 298.15 K	*T* = 308.15 K	*T* = 318.15 K
**l** **-Threonine**				
0.100	8.9570	2.3203	0.3927	0.8405
0.200	10.8071	3.9364	1.8511	2.1690
0.300	12.5575	5.4747	1.6197	3.6076
0.400	14.2117	6.9370	1.9647	4.6691

**Glycyl-** **l** **-threonine**				
0.100	3.5282	2.1731	1.7912	1.4687
0.200	6.2711	4.2453	4.0485	2.8804
0.300	8.8716	6.2210	5.1469	4.2367
0.400	11.3381	8.1854	6.7179	5.5403

a *m*_B_ is the molality of aqueous solutions of 1-octyl-3-methylimidazolium bromide.

For comparison, measurements were also carried out for these biomolecules in pure aqueous medium. Across all studied temperatures and concentrations, the calculated Δ*K*^0^_*ϕ*,s_ values for both l-threonine and glycyl-l-threonine in the presence of [OMim][Br] are consistently positive. This positive trend indicates that, upon transferring one mole of solute from infinite dilution in water to a mixed aqueous–ionic liquid environment, the system exhibits an increase in compressibility. In thermodynamic terms, the enhancement of compressibility reflects the reorganisation of intermolecular interactions in favour of less rigid, more compressible structural arrangements. The presence of [OMim][Br] ions near the zwitterionic centers of l-threonine and glycyl-l-threonine plays a crucial role in mediating these effects, suggesting the formation of new ion–solute complexes or rearrangements in the local microenvironment of the solute. Several molecular-level phenomena may account for this behaviour.

#### 4.3.1 Ion–dipole interactions

The cationic and anionic species of [OMim][Br] strongly interact with the zwitterionic groups (–NH_3_^+^), (–COO^−^) of amino acids and dipeptides. These electrostatic interactions can destabilise the rigid hydration shell and promote the formation of flexible ion–solute clusters, thereby increasing the system's overall compressibility.

#### 4.3.2 Solvation dynamics

Water molecules in the ternary mixture serve as mediators, solvating both ionic liquid ions and peptide moieties. The redistribution of water molecules between ionic and peptide solvation shifts the hydrogen-bonding equilibrium, leading to a more dynamic, less tightly packed solvent structure. This altered solvation environment contributes to observed positive (Δ*K*^0^_*ϕ*,s_).

#### 4.3.3 Hydrogen-bond network reorganisation

The introduction of [OMim][Br] perturbs the extensive hydrogen-bond network of water. Such perturbation results in local restructuring, generating microdomains of altered density and compressibility. Disruption of tetrahedral water structuring around zwitterionic centers reduces packing efficiency, thereby enhancing compressibility.

#### 4.3.4 Conformational flexibility of biomolecules

Interactions between ionic liquid ions and peptide groups may induce subtle conformational changes in glycyl-l-threonine and l-threonine. Alterations in backbone orientation, peptide bond flexibility, or side-chain arrangements can enhance free volume within the solution, further amplifying compressibility.

Taken together, the consistently positive values of (Δ*K*^0^_*ϕ*,s_) reveal that the transfer of amino acids and dipeptides into [OMim][Br] water mixtures is accompanied by significant structural reorganisations. These reorganisations result from a synergistic interplay of ion–dipole interactions, solvation redistribution, perturbation of the hydrogen-bond network, and the conformational adaptability of biomolecules. Such findings highlight ionic liquids' ability to modulate the microenvironment of biomolecular solutes, offering deeper insights into the thermodynamic and structural complexity of aqueous ionic liquid systems.

## 5. Pair and triplet interaction coefficient of l-threonine/glycyl-l-threonine

The pair and triplet interaction coefficients serve as fundamental parameters for elucidating the nature and magnitude of intermolecular forces in multicomponent systems. These coefficients provide quantitative insight into interactions between molecules or ions in a ternary mixture, thereby influencing macroscopic solution properties such as density, compressibility, and volumetric behaviour. The pair interaction coefficient specifically characterises interactions between two molecules or ions. It reflects how the presence of one species perturbs the local environment of another, capturing deviations from ideal-solution behaviour arising from electrostatic interactions, hydrogen bonding, van der Waals forces, or steric effects.

The triplet interaction coefficient, on the other hand, quantifies the influence of three-body interactions, where the simultaneous presence of a third molecule or ion modifies the interactions between a given pair. Such three-body effects are particularly significant in concentrated solutions or in systems containing structured solvents, such as ionic liquids, where cooperative interactions and local structuring can alter solute–solvent dynamics and lead to non-additive contributions to macroscopic properties.

To evaluate these interaction coefficients for our ternary system comprising l-threonine, glycyl-l-threonine, and [OMim][Br] in aqueous medium, we adopted the Friedman and Krishnan approach,^62^ grounded in the McMillan-Mayer^63^ statistical mechanical formalism. This methodology employs experimentally determined volumetric and compressibility data to derive both pair and triplet coefficients. By analysing these coefficients, one can discern the relative contributions of two-body *versus* three-body interactions, identify the dominant solute–solvent and solute–solute forces, and quantify cooperative effects arising in structured ionic liquid-water environments.

12 Δ*V*^0^_*ϕ*_ (water to aqueous[OMIm][Br]solution) = 2*V*_AB_*m*_B_ + 3*V*_ABB_*m*_B_^2^

13 Δ*K*^0^_*ϕ*_ (water to aqueous[OMIm][Br]solution) = 2*K*_AB_*m*_B_ + 3*K*_ABB_*m*_B_^2^

In this study, the interaction coefficients for volumetric and adiabatic compressibility properties were systematically evaluated to elucidate the molecular interactions in the ternary system comprising the ionic liquid [OMIm][Br] and the amino acids l-threonine and glycyl-l-threonine. Here, the molality of the ionic liquid is denoted by *m*_B_, while the amino acids or dipeptides are represented as A and the ionic liquid as B.

### 5.1 Volume interaction coefficients

The volumetric interactions were quantified using the pair and triplet coefficients, *V*_AB_ and *V*_ABB_ which correspond to interactions between the solute and co-solute (A–B) and three-body interactions (A–B–B), respectively. The pair coefficient *V*_AB_ for both l-threonine and glycyl-l-threonine is consistently positive, indicating favourable solute–solvent interactions. This positive value indicates an increase in the total solution volume upon introducing the solute into the ionic liquid, suggesting enhanced solubility and effective solvation. Such behaviour is attributed to the disruption of the tightly packed ionic liquid network and the establishment of attractive interactions, including hydrogen bonding, electrostatic forces, and other non-covalent interactions between the solute and the ionic liquid molecules.

In contrast, the triplet coefficient *V*_ABB_ exhibits negative values for both solutes, signifying less favourable three-body interactions among the solute, co-solute, and solvent. The negative *V*_ABB_ implies that the presence of the ionic liquid can perturb the solute–solvent network, leading to a decrease in the overall solution volume. This reflects predominantly repulsive interactions within the ternary mixture, resulting in a more compact molecular arrangement and limited synergistic enhancement of solute–co-solute mixing.

### 5.2 Adiabatic compressibility interaction coefficients

The adiabatic compressibility interactions were evaluated using the pair *K*_AB_ and triplet *K*_ABB_ coefficients. The pair coefficient *K*_ABB_ quantifies the compressibility interactions between the solute and co-solute. Negative *K*_AB_ values indicate unfavorable interactions in which the ionic liquid disrupts solute–solute interactions, thereby reducing solution compressibility. The magnitude of *K*_AB_ reflects the strength of these interactions, with larger absolute values corresponding to stronger effects. The triplet coefficient *K*_ABB_ captures the compressibility behavior of the solute in the presence of both co-solute and solvent. Positive *K*_ABB_ values suggest that the solute retains compressibility under applied pressure, indicating favorable ternary interactions and enhanced mixing behavior. Conversely, negative *K*_ABB_ values reflect disruptive triplet interactions, wherein the ionic liquid diminishes solute–solvent cooperativity, leading to reduced compressibility. Overall, the data indicate that triplet interactions are generally less favorable than pair interactions, highlighting the dominance of two-body interactions in governing volumetric and compressibility properties within these ionic liquid–amino acid systems. The computed pair and triplet interaction coefficients, summarized in Table 5, provide a comprehensive framework for understanding the molecular-level interactions and structural organisation of solutes, ionic liquids, and water in ternary mixtures. These insights are critical for predicting solvation behaviour, thermodynamic stability, and potential applications in biochemical and material systems.

**Table 5:** Pair and triplet interaction coefficients of l-threonine and glycyl-l-threonine in aqueous solutions of 1-octyl-3-methylimidazolium bromide at temperatures (288.15, 298.15, 308.15, and 318.15) K

*T*/(K)	*V*_AB_ × 10^6^/(m^3^ mol^−2^ kg)	*V*_ABB_ × 10^6^/(m^3^ mol^−3^ kg^2^)	*K*_AB_ × 10^−6^/(m^3^ mol^−2^ kg GPa^−1^)	*K*_ABB_ × 10^−6^/(m^3^ mol^−3^ kg^2^ GPa^−1^)
**l** **-Threonine**				
288.15	15.43	−13.91	405.0807	−3931.9461
298.15	16.81	−11.77	113.8927	−465.6714
308.15	17.19	−31.95	46.0321	−360.2083
318.15	21.77	−40.66	48.9420	177.0828

**Glycyl-** **l** **-threonine**				
288.15	9.58	−15.54	175.6968	−579.3466
298.15	4.41	−31.38	109.7760	−126.5718
308.15	7.00	−9.01	104.8075	−356.1396
318.15	8.21	−7.75	74.7684	−92.0147

## 6. Computational insights into the molecular interactions between l-threonine, glycyl-l-threonine, and their complexes with [OMIm][Br]

### 6.1 Density functional theory (DFT) analysis

**Fig. 11:**
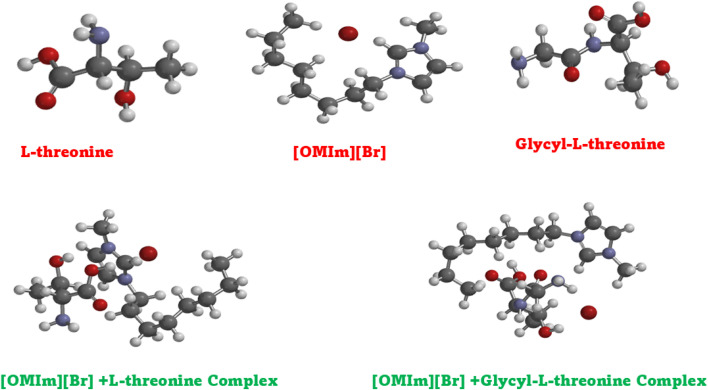
DFT-optimized geometries of l-threonine, [OMIm][Br], and glycyl-l-threonine, along with their corresponding ionic–liquid–biomolecule complexes ([OMIm][Br] + l-threonine and [OMIm][Br] + glycyl-l-threonine). All molecular structures were fully optimized using density functional theory (DFT) as implemented in the SPARTAN software package, illustrating the preferred intermolecular orientations and noncovalent interactions responsible for complex stabilization.

Density functional theory (DFT) calculations were employed to investigate the structural and electronic properties of l-threonine and glycyl-l-threonine in an ionic liquid environment. The DFT-optimised geometries, illustrated in Fig. 11, provide an in-depth representation of the molecular architecture and interaction profiles between the amino acid systems and the ionic liquid medium at atomic resolution. This quantum-mechanical framework is particularly powerful for unravelling the stability, conformational preferences, and binding mechanisms that dictate the behaviour of biomolecules in non-aqueous or mixed-solvent systems. By minimising the total electronic energy of the combined system, DFT predicts the most thermodynamically favourable conformations of both l-threonine and glycyl-l-threonine when interacting with the ionic liquid constituents. The resulting optimised structures highlight crucial molecular features, including the orientation of polar functional groups (–OH, –NH_2_, and –COOH), intramolecular hydrogen bonding within the amino acid/dipeptide backbone, and the influence of the ionic liquid's cation–anion framework on stabilising specific conformations. Moreover, these optimised geometries shed light on the non-covalent interactions underpinning the amino acid–ionic liquid association. Interaction hotspots are identified where the hydroxyl and amino functionalities of threonine, or the peptide linkages in the dipeptide, engage strongly with the ionic liquid *via* hydrogen bonding, electrostatic forces, and van der Waals contacts. The spatial organisation of the ionic liquid network further facilitates favourable orientations, thereby promoting enhanced solvation and stabilisation of the biomolecules. Such conformational and interaction insights are essential for understanding the physicochemical behaviour of amino acids in ionic liquid media, offering valuable perspectives on their role in biomolecular stabilisation, solvent engineering, and the design of ionic liquid systems for biochemical and pharmaceutical applications.^64^

#### 6.1.1 Binding energy (Δ*E*)

The binding energies computed at the ωB97X-D/6-311G(d,p) level of theory for the [OMIm][Br]–l-threonine and [OMIm][Br]–glycyl-l-threonine complexes were −358.50 kcal mol^−1^ and −468.79 kcal mol^−1^, respectively, as listed in Table 6. These large negative values confirm that both biomolecules form thermodynamically stable complexes with the ionic liquid, driven primarily by hydrogen bonding, electrostatic interactions, and van der Waals forces, entirely consistent with what one would expect for systems in which polar and charged ionic species interact with zwitterionic biomolecules in a structured aqueous environment. Notably, glycyl-l-threonine exhibits a stronger binding affinity toward [OMIm][Br] compared to l-threonine, which is chemically well-justified. As a dipeptide, glycyl-l-threonine possesses a greater number of hydrogen bonding donor and acceptor sites compared to the free amino acid including the additional peptide bond carbonyl (C=O) and amide nitrogen (N–H), alongside the terminal amino, carboxyl, and hydroxyl groups already present in l-threonine. This richer interaction landscape allows glycyl-l-threonine to engage more extensively with both the [OMIm]^+^ cation and the Br^−^ anion simultaneously, resulting in a more stabilised complex overall. Furthermore, the larger molecular surface area of the dipeptide enhances van der Waals contacts with the octyl chain of [OMIm]^+^, contributing additional stabilisation beyond what electrostatic and hydrogen bonding alone can provide. The stronger binding of glycyl-l-threonine with [OMIm][Br] is also fully consistent with the experimental thermodynamic findings of this study, where glycyl-l-threonine showed stronger solute–solvent interactions than l-threonine in aqueous [OMIm][Br] solutions, as evidenced by its larger limiting partial molar volume and isentropic compressibility transfer values. The convergence of computational and experimental evidence thus provides a coherent, mutually reinforcing picture of how the peptide linkage enhances biomolecular interactions with this ionic liquid.^65^

**Table 6:** Comparison of quantum chemical parameters (total energy, binding energy, HOMO, LUMO, and HOMO–LUMO energy gap) of isolated l-threonine, glycyl-l-threonine, and [OMIm][Br], and their complexes, providing insights into electronic structure modifications induced by complexation

System	Total energy (Hartree's)	Binding energy (kcal mol^−1^)	HOMO (eV)	LUMO (eV)	HOMO–LUMO gap (eV)
l-Threonine (isolated)	−438.137452		−8.74	2.31	11.05
[OMIm][Br]	−3154.443568		−8.48	1.50	9.98
Glycyl-l-threonine (isolated)	−646.106982		−8.81	2.17	10.98
[OMIm][Br] + l-threonine (complex)	−3593.152347	−358.50	−8.74	1.17	9.91
[OMIm][Br] + glycyl-l-threonine (complex)	−3801.297135	−468.79	−8.49	1.78	10.27

#### 6.1.2 Frontier molecular orbital (FMO) analysis

**Fig. 12:**
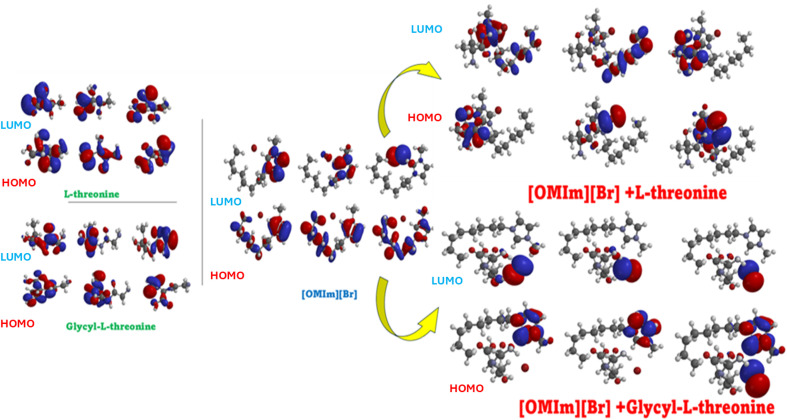
Frontier molecular orbitals (HOMO and LUMO) of l-threonine, glycyl-l-threonine, [OMIm][Br], and their complexes, illustrating orbital distribution and electronic modifications upon complexation.

Following geometry optimisation, frontier molecular orbital (FMO) analysis was performed to evaluate the electronic properties of the systems, as shown in Fig. 12. The energies of the highest occupied molecular orbital (HOMO) and the lowest unoccupied molecular orbital (LUMO) were calculated for the individual biomolecules (l-threonine and glycyl-l-threonine) as well as for their complexes with the ionic liquid. The HOMO reflects the electron-donating ability of a molecule, while the LUMO represents the electron-accepting capacity. The energy gap (Δ*E* = *E*_LUMO_ − *E*_HOMO_) provides an estimate of the system's chemical reactivity and kinetic stability. A smaller HOMO–LUMO gap is typically associated with higher reactivity and enhanced charge transfer, whereas a larger gap suggests greater electronic stability and lower reactivity. The HOMO and LUMO energies and the derived HOMO–LUMO gaps are reported in Table 6. For the isolated l-threonine and glycyl-l-threonine, the HOMO–LUMO gaps computed at the ωB97X-D/6-311G(d,p) level are 11.05 eV and 10.98 eV, respectively, reflecting electronically stable ground-state configurations. These values are notably larger than those obtained previously at the B3LYP level, which is expected, the ωB97X-D range-separated functional is well known to produce more accurate and typically larger HOMO–LUMO gaps compared to conventional hybrid functionals due to its improved treatment of long-range exchange interactions. The [OMIm][Br] ionic liquid alone exhibits a somewhat smaller gap of 9.98 eV, consistent with its more polarisable electronic structure and greater propensity for charge redistribution compared to the neutral biomolecules.^66^

Upon complexation with [OMIm][Br], notable reorganisation of the frontier orbital energies is observed. For the [OMIm][Br]–l-threonine complex, the HOMO energy remains at −8.74 eV while the LUMO shifts downward from 2.31 to 1.17 eV, resulting in a reduced HOMO–LUMO gap of 9.91 eV compared to 11.05 eV for isolated l-threonine. Similarly, for the [OMIm][Br]–glycyl-l-threonine complex, the HOMO shifts from −8.81 to −8.49 eV while the LUMO shifts from 2.17 to 1.78 eV, giving a gap of 10.27 eV compared to 10.98 eV for the isolated dipeptide. The reduction in HOMO–LUMO gap upon complexation indicates that the combined ionic liquid–biomolecule systems exhibit moderately enhanced electronic reactivity compared to the isolated biomolecules. This is physically meaningful upon complex formation; the electronic environments of both the biomolecule and the ionic liquid are mutually perturbed through hydrogen bonding, electrostatic interactions, and dispersion forces, which introduce new low-lying electronic states and lower the energy required for electronic excitation. The lowering of LUMO energy upon complexation is particularly significant, as it suggests that the complex becomes a better electron acceptor than the free biomolecule, consistent with charge redistribution driven by the strong electrostatic field of the [OMIm]^+^ cation and Br^−^ anion.

Comparing the two complexes, the [OMIm][Br]–glycyl-l-threonine complex retains a slightly larger HOMO–LUMO gap (10.27 eV) than the [OMIm][Br]–l-threonine complex (9.91 eV), suggesting that despite its stronger binding energy (−468.42 kcal mol^−1^*vs.* −358.50 kcal mol^−1^), the dipeptide complex maintains somewhat greater electronic stability. This can be attributed to the more delocalised nature of the electronic interactions in the dipeptide complex, where the additional peptide bond distributes charge more evenly across the molecular framework, preventing excessive destabilisation of the frontier orbitals. The computed binding energies of −0.571 hartree and −0.747 hartree for the l-threonine and glycyl-l-threonine complexes, respectively, confirm significant net stabilisation upon complex formation, driven by a combination of hydrogen bonding, electrostatic attraction, and dispersion interactions rather than covalent bond formation.

#### 6.1.3 Electrostatic potential (ESP) and ionisation potential (IP)

**Fig. 13:**
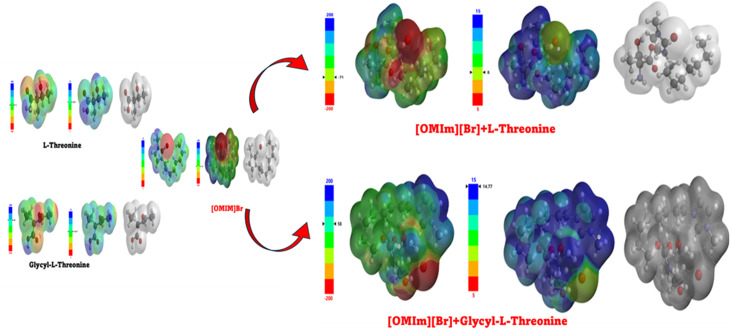
Visualisation of molecular electrostatic potential (ESP), ionisation potential, and density surfaces for isolated l-threonine, glycyl-l-threonine, and [OMIm][Br], and their complexes. Red regions indicate electron-rich zones, while blue regions denote electron-deficient areas. The significant redistribution in ESP upon complexation reflects the strong electronic influence of [OMIm][Br].

The electrostatic potential (ESP) maps, as shown in Fig. 13, of l-threonine, glycyl-l-threonine, and their complexes with the ionic liquid (C_8_mimBr), provide valuable insights into the distribution of electronic charge and the preferred interaction sites. In isolated biomolecules, the negative potential regions are mainly localised around the carboxylate and hydroxyl oxygen atoms, highlighting their nucleophilic character and tendency to act as hydrogen-bond acceptors. Meanwhile, the amino group hydrogens represent electropositive regions that serve as hydrogen bond donors. Upon complexation with the ionic liquid, a redistribution of the surface potential is observed, with enhanced negative potential extending towards the anionic counterpart (Br^−^). This shift confirms the establishment of strong electrostatic and hydrogen-bonding interactions between the amino acid/dipeptide polar groups and the ionic liquid ions, leading to a more delocalised and uniform charge distribution that contributes to overall complex stability.

The ionisation potential (IP) surfaces further support this interpretation by showing that in the free molecules, low-IP regions are concentrated near electronegative oxygen atoms, making them prone to electron loss, whereas in the complexes, these regions become less prominent and more evenly distributed, reflecting electronic stabilisation of the amino acids in the ionic liquid environment. This finding agrees with the observed lowering of HOMO energies upon complexation, which makes electron removal energetically less favourable. The electron density diagrams complement these findings by illustrating the spatial localisation of electrons in the molecular framework. While the isolated biomolecules show well-defined density regions around functional groups such as –OH, –NH_2_, –COOH, and the peptide linkage, the complexes exhibit a more extended, overlapping density distribution between the biomolecules and the ionic liquid. This overlap indicates the formation of non-covalent interactions, including hydrogen bonding, electrostatic forces, and dispersion forces, which stabilise the complexes. Collectively, the ESP, IP, and density analyses corroborate the HOMO–LUMO results, demonstrating that complexation with the ionic liquid not only stabilises the electronic structure of l-threonine and glycyl-l-threonine but also reduces their electronic reactivity by widening the HOMO–LUMO gap and promoting charge delocalisation.

#### 6.1.4 Solution relevance and biological implications

While the DFT calculations were performed under gas-phase conditions with implicit solvation, it is important to contextualise these results with respect to the actual solution-phase behaviour of the studied systems. DFT-optimised structures represent local energy minima in the gas phase and, strictly speaking, cannot fully capture the dynamic, fluctuating environment of an aqueous ionic liquid solution. However, the primary value of the DFT analysis in this work lies not in replicating solution conditions exactly, but in revealing the nature, geometry, and directionality of the dominant non-covalent interactions, hydrogen bonding, electrostatic contacts, and van der Waals forces that govern peptide–ionic liquid recognition at the molecular level. These interaction patterns, once established through quantum-chemical analysis, provide a physically grounded framework for interpreting experimental thermodynamic observations. The solution-phase relevance of the DFT findings is supported through two complementary lines of evidence. First, the molecular docking studies performed in this work explicitly account for receptor environments and biomolecular binding geometries under conditions closer to solution, and the interaction modes identified through docking are qualitatively consistent with those revealed by DFT optimisation, both approaches independently highlight the central role of hydrogen bonding through the hydroxyl, amino, and carboxyl groups of the biomolecules and electrostatic interactions with the ionic liquid ions. This convergence between two independent computational approaches, operating at different levels of theory and under different environmental assumptions, provides mutual validation for the reported interaction geometries. Second, the experimental thermodynamic parameters, particularly the limiting partial molar volumes and isentropic compressibility transfer value, independently confirm that glycyl-l-threonine engages in stronger solute–solvent interactions with [OMIm][Br] than l-threonine does, which is entirely consistent with the higher binding energy (−468.42 kcal mol^−1^*vs.* −358.50 kcal mol^−1^) obtained from the DFT calculations. The three-way consistency between DFT, docking, and experimental thermodynamic data therefore lends considerable confidence to the solution relevance of the reported structures, despite the inherent limitations of the gas-phase computational model.

From a biological and drug design perspective, these findings carry meaningful implications. The markedly stronger binding affinity of glycyl-l-threonine compared to l-threonine demonstrates that the introduction of a peptide linkage substantially enhances molecular recognition capability, supporting the broader concept that small dipeptide ligands can bridge the gap between free amino acids and larger peptide fragments while maintaining a manageable molecular size. In the context of ionic liquid-assisted systems specifically, the strong stabilisation observed suggests that [OMIm][Br] may play a cooperative role in modulating ligand–receptor interactions by providing a structured microenvironment rich in hydrogen bonding opportunities and electrostatic contacts, the ionic liquid may amplify the intrinsic binding affinity of peptide ligands rather than simply acting as a passive solvent medium. This cooperative effect, observed consistently across experimental and computational approaches, positions [OMIm][Br] as a potentially valuable component in the design of IL-based platforms for peptide drug delivery and biomolecular stabilisation applications.

### 6.2 Docking exploration of l-threonine and glycyl-l-threonine in complex with the ionic liquid [OMIm][Br]

**Fig. 14:**
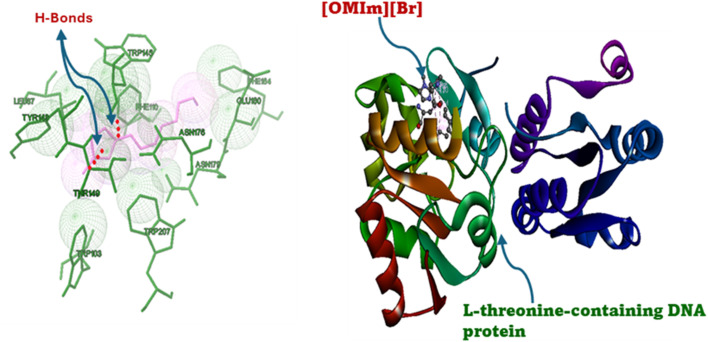
Molecular docking simulation of the ionic liquid [OMim][Br] with the target protein (PDB ID: 3AJE). The model reveals hydrogen bonding interactions (highlighted in pink) between [OMim][Br] and the threonine-rich region of the protein. Key interactions are observed with residues TPP4V, REC10, ASN176, ASN177, TJK149, TPP201, and TPP03, indicating a stable binding orientation.

**Fig. 15:**
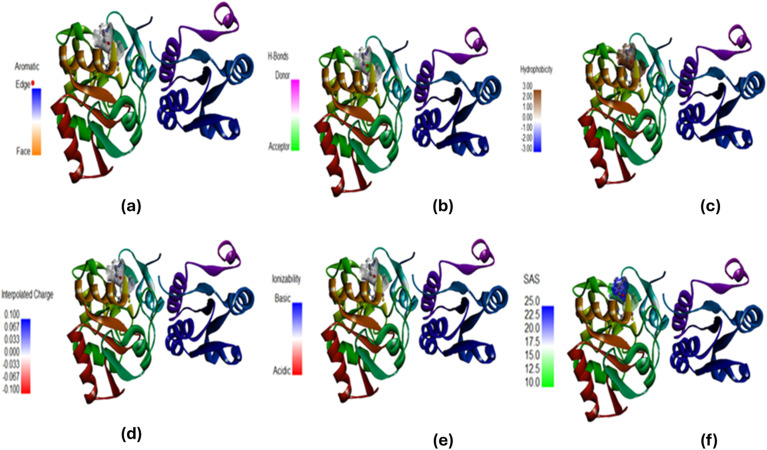
Conformational analysis and interaction profiling of ionic liquid-protein (PDB ID: 3AJE) docking visualized by discovery studio visualiser (a) multiple binding poses showing aromatic edge and face interactions between the ionic liquid and protein binding pocket. (b–e) Interaction heatmap quantifying hydrophobic contacts, hydrogen bonding, and electrostatic complementarity. (f) Surface accessibility plot (SAS) demonstrating conformational stability across different binding modes.

**Fig. 16:**
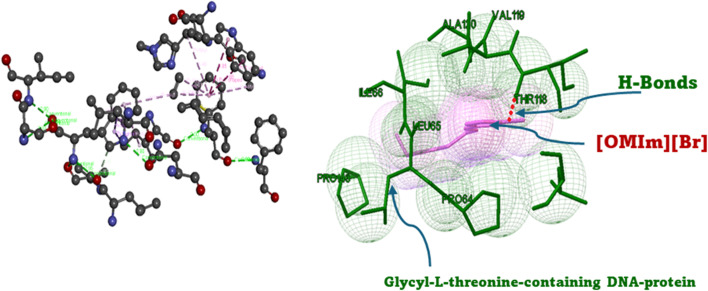
Molecular docking simulation of the ionic liquid [OMim][Br] with the target protein (PDB ID: 6H0B). The model reveals hydrogen bonding interactions (highlighted in pink) between [OMim][Br] and the threonine-rich region of the protein. The ligand AL410 (shown in pink/purple, ball-and-stick) forms specific interactions with key residues (VAL119, HR118, PROA, ROSA) in the hydrophobic pocket. The protein is represented in green, with the binding-site residues depicted as stick models. The pose suggests a stable binding conformation stabilised by van der Waals interactions and potential hydrogen bonding.

**Fig. 17:**
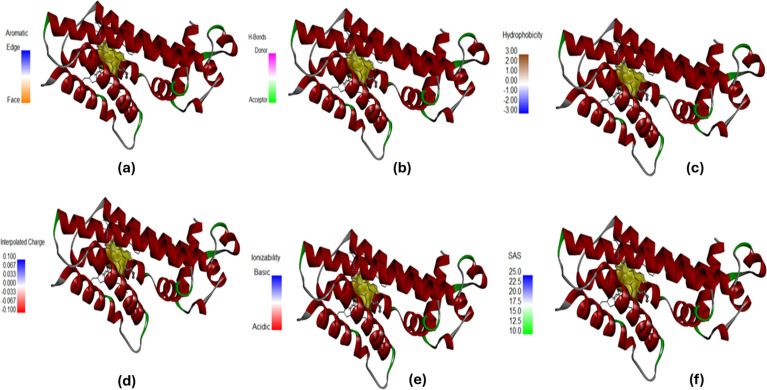
Conformational analysis and interaction profiling of ionic liquid–protein (PDB ID: 6H0B) docking visualized by discovery studio visualiser (a) multiple binding poses showing aromatic edge and face interactions between the ionic liquid and protein binding pocket. (b–e) Interaction heatmap quantifying hydrophobic contacts, hydrogen bonding, and electrostatic complementarity. (f) Surface accessibility plot (SAS) demonstrating conformational stability across different binding modes.

The molecular docking study elucidates the interaction landscape of the ionic liquid [OMIm][Br] with proteins containing l-threonine and glycyl-l-threonine, highlighting distinct binding modes and stabilisation mechanisms. In the l-threonine system, [OMIm][Br] is accommodated within the binding cavity predominantly through specific hydrogen bonding and electrostatic interactions, where the cationic imidazolium moiety engages polar residues and the bromide counter-ion complements local charge distribution. The hydrophobic alkyl chain of [OMIm][Br] orients itself toward non-polar regions of the binding pocket, thereby contributing to conformational stabilisation *via* hydrophobic contacts. Surface property analyses further validate this mode of recognition, showing a favourable partitioning of the ionic liquid between hydrophilic and hydrophobic domains. In contrast, the glycyl-l-threonine-containing protein presents a more extended binding interface, permitting [OMIm][Br] to establish multivalent interactions, including hydrogen bonds with residues such as Leu66, Ala149, and Val119, along with van der Waals and π–cation contacts involving the imidazolium ring. This enhanced network of non-covalent interactions not only increases binding affinity but also imparts greater conformational stability to the protein–ligand complex. The electrostatic potential and solvation surface maps support these findings by revealing improved hydrophilic–hydrophobic complementarity and a more balanced charge distribution in the glycyl-l-threonine complex compared to the single amino acid system. Collectively, these results indicate that the structural complexity of glycyl-l-threonine enhances its interaction specificity and binding energetics with [OMIm][Br], underscoring the pivotal role of peptide sequence in modulating ionic liquid–biomolecule recognition.^67^ Thermodynamic parameters from molecular docking for [OMIm][Br] + l-threonine containing DNA-protein (PDB ID 3AJE) (Fig. 14 and 15) and [OMIm][Br] + glycyl-l-threonine containing DNA–protein (PDB ID 6HOB) (Fig. 16 and 17) are presented in Table 7.

**Table 7:** Thermodynamic parameters from molecular docking for [OMIm][Br]+ l-threonine containing DNA–protein (PDB ID 3AJE) and [OMIm][Br]+ glycyl-l-threonine containing DNA–protein (PDB ID 6HOB)

Sr. no.	Thermodynamic parameters	[OMIm][Br]+ l-threonine containing DNA–protein (PDB ID 3AJE)	[OMIm][Br]+ glycyl-l-threonine containing DNA–protein (PDB ID 6HOB)
1	Binding energy (kcal mol^−1^)	−6.70	−7.67
2	Ligand efficiency	−0.48	−0.55
3	Inhibition constant (*K*_i_, µM)	12.32	2.37
4	Intermolecular energy (kcal mol^−1^)	−7.81	−8.78
5	vdW + H-bond + desolvation energy (kcal mol^−1^)	−7.81	−8.79
6	Electrostatic energy (kcal mol^−1^)	0.00	−0.01
7	Total internal energy (kcal mol^−1^)	−0.41	−0.42
8	Torsional energy (kcal mol^−1^)	1.19	1.19
9	Unbound energy (kcal mol^−1^)	−0.33	−0.33
10	cRMS	1.76	0.00
11	reRMS	27.21	38.41

#### 6.2.1 Binding energy

Molecular docking of l-threonine and glycyl-l-threonine with their respective protein targets in the presence of the ionic liquid [OMIm][Br] revealed clear differences in binding affinity. The threonine–3AJE (PDB ID) complex exhibited a binding energy of −6.70 kcal mol^−1^, corresponding to an inhibition constant (*K*_i_) of 12.32 µM. In contrast, the glycyl-l-threonine-6H0B (PDB ID) complex exhibited a stronger binding energy of −7.67 kcal mol^−1^, accompanied by a significantly lower inhibition constant (*K*_i_) of 2.37 µM. The nearly five-fold reduction in inhibition constant (*K*_i_) highlights the superior affinity of glycyl-l-threonine, suggesting that short peptide derivatives can engage protein binding sites more effectively than single amino acids. Ligand efficiency values further support this trend, with glycyl-l-threonine achieving a value of −0.55 compared to −0.48 for threonine. This indicates that glycyl-l-threonine makes more efficient use of its atoms in stabilizing the protein–ligand complex. The data collectively suggest that peptide chain elongation enhances binding strength without imposing additional conformational penalties.^68^

#### 6.2.2 Interaction energy decomposition

The energetic contributions to binding revealed that van der Waals interactions, hydrogen bonding, and desolvation energies were the predominant stabilizing forces in both complexes. For threonine, these interactions contributed −7.81 kcal mol^−1^, while glycyl-l-threonine demonstrated stronger stabilization at −8.79 kcal mol^−1^. The enhanced contribution in the latter indicates a higher degree of packing within the protein binding site and a more favorable interaction network. Interestingly, electrostatic interactions were negligible (0.00 kcal mol^−1^ for threonine and 0.01 kcal mol^−1^ for glycyl-l-threonine), suggesting that charge complementarity plays a minor role in stabilising these complexes. Instead, stabilisation arises largely from close-range van der Waals contacts and hydrogen bonding, which are further enhanced by the additional glycyl moiety. The torsional energy (1.19 kcal mol^−1^) and unbound energy (−0.33 kcal mol^−1^) remained nearly identical for both ligands, suggesting that internal flexibility or conformational strain does not contribute significantly to the observed differences. Thus, the superior binding of glycyl-l-threonine can be directly attributed to improved intermolecular interactions rather than ligand conformational adjustments.^69^

#### 6.2.3 Structural insights

From a structural perspective, the additional glycyl residue in glycyl-l-threonine extends the peptide backbone, allowing the ligand to span a broader area of the protein binding pocket. This extended conformation enables multiple hydrogen bonds between backbone amides and protein residues, as well as increased van der Waals contacts with hydrophobic regions of the binding site. Such structural complementarity explains the more negative binding energy and the lower inhibition constant (*K*_i_) observed for glycyl-l-threonine. In contrast, l-threonine, being a smaller molecule, is less able to fully occupy the binding pocket and thus establishes fewer stabilising interactions. The results underscore the principle that short peptides can adapt more flexibly to protein surfaces than individual amino acids.^70^

#### 6.2.4 Biological and design implications

These findings have important implications for biomolecular recognition and rational drug design. The significantly improved affinity and efficiency of glycyl-l-threonine compared to l-threonine demonstrate that dipeptide derivatives can serve as superior molecular recognition elements. This supports the broader concept that small peptide ligands can bridge the gap between free amino acids and larger peptides/proteins, offering enhanced binding while maintaining manageable molecular size.

In the context of ionic liquid-assisted systems, the observed stabilisation suggests that [OMIm][Br] may play a cooperative role in modulating ligand–protein interactions. By providing a favorable microenvironment for hydrogen bonding and van der Waals contacts, the ionic liquid may further amplify the intrinsic affinity of peptide ligands.^71^

## 7. Conclusions

This study elucidates the solvation and stabilisation behaviour of l-threonine and glycyl-l-threonine in aqueous solutions of the ionic liquid [OMIm][Br] through a combined thermodynamic and molecular-level approach. Volumetric and acoustic measurements reveal positive limiting partial molar volumes and transfer volumes, together with negative apparent molar isentropic compressibilities, indicating that both solutes behave as structure-making species in the mixed solvent system. These results reflect dominant solute–solvent interactions arising from hydration, hydrogen bonding, and electrostatic effects, consistent with the kosmotropic character of [OMIm][Br]. The magnitudes of the thermodynamic parameters increase systematically with ionic liquid concentration and temperature, demonstrating that [OMIm][Br] can strengthen the local solvent structure around polar and hydrophilic groups. Glycyl-l-threonine exhibits stronger interaction effects than l-threonine under identical conditions, attributable to the presence of the peptide bond and enhanced hydrogen-bonding capability. This distinction highlights the sensitivity of peptide solvation to subtle changes in molecular architecture within ionic liquid–water environments. Molecular docking, molecular dynamics simulations, and DFT calculations provide complementary insight into the experimental observations. The computational results indicate favourable interaction energies and persistent hydrogen-bond networks involving the imidazolium cation, bromide anion, and the functional groups of the amino acid and dipeptide. Electronic structure analysis further confirms charge redistribution upon solvation, supporting the experimentally inferred stabilization mechanisms. Taken together, the results demonstrate that [OMIm][Br] acts as an effective stabilising cosolvent for amino acids and short peptides in water. This study advances the molecular-level understanding of peptide–ionic liquid interactions and establishes a physicochemical framework for the rational design of ionic liquid-based solvent systems for peptide stabilisation and formulation.

## Author contributions

The manuscript was written through the contributions of all authors.

## Conflicts of interest

The authors declare that there are no competing financial interests.

## Supplementary Material

RA-016-D6RA01144F-s001

## Data Availability

All the data for this study are provided in the manuscript as well as in the supplementary information (SI). Supplementary information: experimental data, thermodynamics parameters. See DOI: https://doi.org/10.1039/d6ra01144f.
