# Supplementary material for: Volumetric, acoustic, and computational investigation of l-threonine and glycyl-l-threonine interactions in aqueous 1-octyl-3-methylimidazolium bromide solutions
Source: RSC Adv. 2026 Jun 2;16(33):30026–51. doi: 10.1039/d6ra01144f (PMC13231369; doi:10.1039/d6ra01144f)
Supplement: RA-016-D6RA01144F-s001 [file RA-016-D6RA01144F-s001.pdf]

Supplementary Information (SI) for RSC Advances.
This journal is © The Royal Society of Chemistry 2026

Supporting Information

Volumetric, Acoustic, and Computational Investigation of L-Threonine and Glycyl-L-Threonine Interactions in Aqueous 1-Octyl-3-methylimidazolium Bromide Solutions

Ravinder Sharma[a], Sandeep Kumar[b], Marc Mulamba Tshibangu[b,*], Indra Bahadur[c,*]

[a]Department of Chemistry, National Institute of Technology, Hamirpur–177005

[b]Department of Chemical Engineering, Mangosuthu University of Technology, Jacobs, Durban 4026, South Africa

[c]Department of Chemistry, North-West University (Mafikeng Campus), Private Bag X2046, Mmabatho 2735, South Africa

*Corresponding author e-mail: tshibangu.marc@mut.ac.za; bahadur.indra@nwu.ac.za

Table S1: Profile of chemicals used in the experiment.

Chemicals	CAS No	Source	Molar mass($g \cdot mol^{-1}$)	Purity# Mass Fraction	Structure
L-threonine	72-19-5	Merck, Germany	119.12	≥0.99	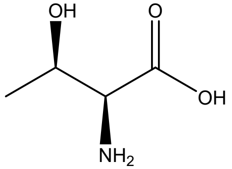
Glycyl-L-threonine	7093-70-1	Merck, Germany	176.17	≥0.99	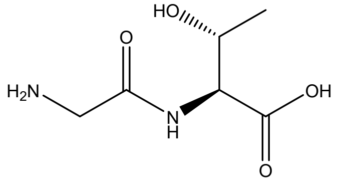
1-methylimidazole	616-47-7	Merck, Germany	82.10	≥0.99	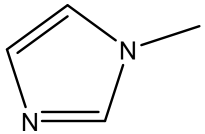
1-bromooctane	111-83-1	Sigma-Aldrich, Germany	193.12	≥0.99	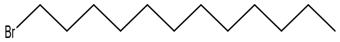
Acetonitrile	75-05-8	LOBA Chemie Pvt. Ltd., Mumbai, India	41.05	≥0.99	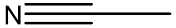

Hexane	110-54-3	TCI Pvt. Ltd	86.18	≥0.99	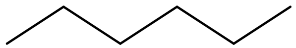
[OMim][Br]	61545-99-1	Synthesized in lab	275.23	≥0.96*	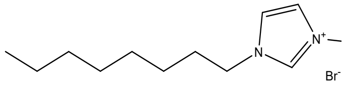

[#]as declared by the supplier, *based on spectroscopic analysis (IR and NMR).

Table S2 Experimental values of ρ and $V\phi$ for L-threonine and Glycyl-L-threonine in water containing [OMim] [Br] determined at (288.15, 298.15, 308.15, and 318.15) K under a pressure of 0.1 MPa.

[a]m_A /(mol·kg^{-1})	$\rho \times 10^{-3}$ / (kg·m^{-3})				$V_\phi \times 10^6$ / (m^3·mol^{-1})			
	T=288.15 K	T=298.15 K	T=308.15 K	T=318.15 K	T=288.15 K	T=298.15 K	T=308.15 K	T=318.15 K
				L-threonine + 0.00 mol·kg^{-1} [OMim] [Br]				
0.00000	0.999130[a]	0.997050[a]	0.994040[a]	0.990220[a]				
0.09947	1.003258	1.001178	0.998168	0.994348	77.33	77.41	77.51	77.65
0.11043	1.003713	1.001633	0.998623	0.994803	77.30	77.37	77.48	77.61
0.20746	1.007740	1.005660	1.002650	0.998830	76.99	77.06	77.17	77.30
0.30817	1.011919	1.009839	1.006829	1.003009	76.67	76.74	76.85	76.98
0.39894	1.015686	1.013606	1.010596	1.006776	76.39	76.46	76.56	76.69
0.49718	1.019763	1.017683	1.014673	1.010853	76.08	76.15	76.25	76.38
0.60278	1.024145	1.022065	1.019055	1.015235	75.75	75.82	75.92	76.05
0.69235	1.027863	1.025783	1.022773	1.018953	75.48	75.55	75.65	75.77

0.80693	1.032618	1.030538	1.027528	1.023708	75.13	75.20	75.30	75.42
0.98174	1.039872	1.037792	1.034782	1.030962	74.61	74.68	74.77	74.89
L-threonine + 0.10 mol·kg^{-1} [OMim] [Br]								
0.00000	1.004330[a]	1.001550[a]	0.997980[a]	0.993620[a]				
0.10861	1.008783	1.006003	1.002433	0.998073	77.62	77.72	77.85	78.01
0.19873	1.012478	1.009698	1.006128	1.001768	77.33	77.43	77.56	77.72
0.30237	1.016727	1.013947	1.010377	1.006017	77.01	77.11	77.24	77.39
0.41892	1.021506	1.018726	1.015156	1.010796	76.65	76.75	76.87	77.03
0.50745	1.025135	1.022355	1.018785	1.014425	76.38	76.47	76.60	76.75
0.61858	1.029692	1.026912	1.023342	1.018982	76.04	76.13	76.26	76.41
0.70929	1.033411	1.030631	1.027061	1.022701	75.77	75.86	75.98	76.13
0.79564	1.036951	1.034171	1.030601	1.026241	75.51	75.60	75.72	75.87
0.91483	1.041838	1.039058	1.035488	1.031128	75.15	75.24	75.36	75.51
0.99742	1.045224	1.042444	1.038874	1.034514	74.91	75.00	75.12	75.26
L-threonine + 0.20 mol·kg^{-1} [OMim] [Br]								
0.00000	1.009530[a]	1.006050[a]	1.001920[a]	0.997020[a]				
0.11042	1.014002	1.010522	1.006392	1.001492	77.91	78.04	78.20	78.38
0.21349	1.018176	1.014696	1.010566	1.005666	77.59	77.72	77.87	78.06
0.29738	1.021574	1.018094	1.013964	1.009064	77.33	77.46	77.61	77.79
0.39875	1.025679	1.022199	1.018069	1.013169	77.02	77.15	77.30	77.48
0.51217	1.030273	1.026793	1.022663	1.017763	76.68	76.81	76.95	77.13

0.59893	1.033787	1.030307	1.026177	1.021277	76.42	76.54	76.69	76.86
0.71081	1.038318	1.034838	1.030708	1.025808	76.09	76.21	76.35	76.52
0.80736	1.042228	1.038748	1.034618	1.029718	75.80	75.92	76.06	76.23
0.89872	1.045928	1.042448	1.038318	1.033418	75.53	75.65	75.79	75.96
0.98724	1.049513	1.046033	1.041903	1.037003	75.28	75.39	75.53	75.70
L-threonine + 0.30 mol·kg^{-1} [OMim] [Br]								
0.00000	1.014730[a]	1.01055[a]	1.005860[a]	1.000420[a]				
0.10847	1.019069	1.01489	1.010416	1.004737	78.21	78.37	78.63	78.96
0.19879	1.022682	1.01850	1.014209	1.008332	77.93	78.09	78.33	78.68
0.30523	1.026939	1.02276	1.018680	1.012568	77.61	77.77	77.99	78.35
0.39638	1.030585	1.02641	1.022508	1.016196	77.34	77.49	77.70	78.07
0.49891	1.034686	1.03051	1.026814	1.020277	77.03	77.18	77.37	77.76
0.61325	1.039260	1.03508	1.031617	1.024827	76.69	76.84	77.01	77.41
0.70566	1.042956	1.03878	1.035498	1.028505	76.42	76.57	76.72	77.14
0.79943	1.046707	1.04253	1.039436	1.032237	76.14	76.29	76.43	76.86
0.89184	1.050404	1.04622	1.043317	1.035915	75.88	76.02	76.15	76.59
0.99157	1.054393	1.05021	1.047506	1.039884	75.59	75.73	75.84	76.29
L-threonine + 0.40 mol·kg^{-1} [OMim] [Br]								
0.00000	1.019930[a]	1.015050[a]	1.009800[a]	1.003820[a]				
0.10215	1.023965	1.019085	1.014172	1.007855	78.51	78.70	79.02	79.15
0.21687	1.028496	1.023616	1.019082	1.012386	78.16	78.36	78.64	78.79

0.30721	1.032065	1.027185	1.022949	1.015955	77.89	78.08	78.34	78.52
0.41098	1.036164	1.031284	1.027390	1.020054	77.59	77.77	78.00	78.20
0.50765	1.039982	1.035102	1.031527	1.023872	77.30	77.49	77.69	77.91
0.61438	1.044198	1.039318	1.036095	1.028088	76.99	77.17	77.35	77.59
0.71479	1.048164	1.043284	1.040393	1.032054	76.70	76.88	77.03	77.29
0.81053	1.051946	1.047066	1.044491	1.035836	76.42	76.60	76.73	77.01
0.89732	1.055374	1.050494	1.048205	1.039264	76.17	76.35	76.45	76.76
0.99084	1.059068	1.054188	1.052208	1.042958	75.91	76.08	76.16	76.48
Glycyl-L-threonine + 0.00 mol·kg^{-1} [OMim] [Br]								
0.00000	0.999130	0.997050	0.994040	0.990220				
0.01042	0.999839	0.997753	0.994740	0.990918	108.13	108.71	109.14	109.50
0.02015	1.000500	0.998410	0.995394	0.991570	108.06	108.64	109.07	109.43
0.02997	1.001168	0.999073	0.996054	0.992228	107.98	108.57	109.00	109.36
0.04069	1.001897	0.999797	0.996774	0.992946	107.91	108.49	108.92	109.28
0.04984	1.002519	1.000414	0.997389	0.993559	107.84	108.43	108.85	109.21
0.05981	1.003197	1.001087	0.998059	0.994227	107.77	108.35	108.78	109.14
0.07034	1.003913	1.001798	0.998767	0.994933	107.69	108.28	108.70	109.06
0.08079	1.004624	1.002503	0.999469	0.995633	107.61	108.20	108.62	108.98
0.08987	1.005241	1.003116	1.000079	0.996241	107.55	108.13	108.56	108.92
0.09843	1.005823	1.003694	1.000654	0.996815	107.48	108.07	108.50	108.85
Glycyl-L-threonine + 0.10 mol·kg^{-1} [OMim] [Br]								

0.00000	1.004330	1.001550	0.997980	0.993620				
0.01085	1.005063	1.002279	0.998706	0.994344	108.31	108.83	109.28	109.66
0.02037	1.005707	1.002919	0.999343	0.994979	108.24	108.76	109.21	109.59
0.03116	1.006436	1.003644	1.000065	0.995698	108.17	108.68	109.13	109.51
0.04025	1.007051	1.004255	1.000673	0.996305	108.10	108.61	109.06	109.45
0.04938	1.007668	1.004868	1.001284	0.996914	108.03	108.55	108.99	109.38
0.06013	1.008395	1.005591	1.002003	0.997631	107.96	108.47	108.92	109.30
0.06908	1.009000	1.006192	1.002601	0.998228	107.89	108.40	108.85	109.24
0.08143	1.009835	1.007022	1.003428	0.999051	107.80	108.31	108.76	109.15
0.09071	1.010462	1.007646	1.004048	0.999670	107.73	108.25	108.69	109.08
0.10726	1.011581	1.008758	1.005156	1.000774	107.62	108.13	108.57	108.96
Glycyl-L-threonine + 0.20 mol·kg^{-1} [OMim][Br]								
0.00000	1.009530	1.006050	1.001920	0.997020				
0.01034	1.010225	1.006742	1.002609	0.997707	108.50	108.94	109.41	109.82
0.02061	1.010915	1.007429	1.003293	0.998389	108.42	108.86	109.34	109.75
0.02978	1.011531	1.008042	1.003903	0.998997	108.35	108.80	109.27	109.68
0.04034	1.012241	1.008749	1.004607	0.999699	108.28	108.72	109.19	109.60
0.04937	1.012848	1.009353	1.005208	1.000298	108.21	108.66	109.13	109.54
0.06039	1.013588	1.010090	1.005942	1.001030	108.14	108.58	109.05	109.46
0.07142	1.014329	1.010828	1.006677	1.001762	108.06	108.50	108.97	109.38
0.08072	1.014954	1.011450	1.007296	1.002380	107.99	108.43	108.90	109.31

0.08976	1.015562	1.012055	1.007898	1.002980	107.92	108.37	108.84	109.25
0.09971	1.016231	1.012721	1.008561	1.003641	107.85	108.29	108.77	109.17
Glycyl-L-threonine + 0.30 mol·kg^{-1} [OMim] [Br]								
0.00000	1.014730	1.010550	1.005860	1.000420				
0.01135	1.015488	1.011306	1.006613	1.001170	108.66	109.03	109.53	109.97
0.01989	1.016059	1.011875	1.007179	1.001735	108.60	108.97	109.47	109.91
0.02976	1.016718	1.012532	1.007833	1.002387	108.53	108.90	109.40	109.84
0.04015	1.017412	1.013224	1.008522	1.003074	108.45	108.83	109.32	109.76
0.05031	1.018091	1.013901	1.009196	1.003745	108.38	108.75	109.25	109.69
0.06173	1.018854	1.014661	1.009953	1.004500	108.30	108.67	109.17	109.60
0.07124	1.019489	1.015295	1.010583	1.005129	108.23	108.60	109.10	109.54
0.08019	1.020087	1.015891	1.011177	1.005721	108.17	108.54	109.04	109.47
0.09375	1.020993	1.016794	1.012076	1.006617	108.07	108.44	108.94	109.37
0.09921	1.021357	1.017157	1.012438	1.006978	108.03	108.41	108.90	109.33
Glycyl-L-threonine + 0.40 mol·kg^{-1} [OMim] [Br]								
0.00000	1.019930	1.015050	1.009800	1.003820				
0.01147	1.020692	1.015809	1.010557	1.004575	108.82	109.22	109.65	110.12
0.02058	1.021297	1.016412	1.011158	1.005174	108.75	109.16	109.59	110.05
0.02935	1.021879	1.016993	1.011737	1.005751	108.69	109.10	109.53	109.99
0.03981	1.022573	1.017685	1.012427	1.006439	108.62	109.02	109.45	109.91
0.05125	1.023333	1.018443	1.013183	1.007192	108.54	108.94	109.37	109.83

0.06084	1.023970	1.019078	1.013815	1.007823	108.47	108.87	109.30	109.76
0.06921	1.024526	1.019632	1.014368	1.008374	108.41	108.82	109.24	109.70
0.08037	1.025267	1.020370	1.015104	1.009108	108.33	108.74	109.16	109.62
0.09034	1.025929	1.021031	1.015762	1.009764	108.26	108.67	109.09	109.55
0.10275	1.026753	1.021852	1.016582	1.010581	108.17	108.58	109.00	109.46

[a]m_A is the molality of amino acids/ dipeptides in aqueous solutions of 1-octyl-3-methylimidazolium bromide. [a] repreents a reference (53) from where density values have been taken. Standard uncertainties u obtained are u (ρ) = 5 × 10^{-3} kg·m^{-3}; u (m) = 2 × 10^{-5} mol·kg^{-1}; u (T) = 0.001 K and u (p) = 0.01 MPa.

Table S3 Measurements of the limiting apparent molar volume (V_ϕ^0) and experimental slope (S_v^*) for L-threonine and Glycyl-L-threonine carried out in aqueous solutions of 1-octyl-3-methylimidazolium bromide across temperatures of 288.15 to 318.15 K at an experimental pressure of , p = 0.1 MPa

[a]m_B / (mol·kg^{-1})	288.15 K	298.15 K	308.15 K	318.15 K
		L-threonine		
		$V_\phi^0 \times 10^6$ / (m^3·mol^{-1})		
0.000	77.63(±0.0063)	77.70(±0.0063)	77.81(±0.0064)	77.95(±0.0064)
0.100	77.93(±0.0067)	78.03(±0.0067)	78.17(±0.0068)	78.33(±0.0063)
0.200	78.23(±0.0062)	78.36(±0.0063)	78.52(±0.0063)	78.71(±0.0064)

0.300	78.52(±0.0061)	78.68(±0.0062)	78.95(±0.0069)	79.28(±0.0063)
0.400	78.80(±0.0060)	78.99(±0.0061)	79.33(±0.0070)	79.44(±0.0062)
		$S_V^* \times 10^6$ / (m^3·kg·mol^{-2})		
0.000	-3.09(±0.0115)	-3.10(±0.0115)	-3.12(±0.0116)	-3.13(±0.0117)
0.100	-3.04(±0.0107)	-3.06(±0.0108)	-3.07(±0.0109)	-3.09(±0.0110)
0.200	-3.01(±0.0100)	-3.02(±0.0101)	-3.04(±0.0102)	-3.06(±0.0103)
0.300	-2.97(±0.0099)	-2.99(±0.0111)	-3.15(±0.0101)	-3.02(±0.0102)
0.400	-2.93(±0.0097)	-2.95(±0.0098)	-3.21(±0.0116)	-3.00(±0.0100)
		Glycyl-L-threonine		
		$V_\phi^0 \times 10^6$ / (m^3·mol^{-1})		
0.000	108.20(±0.0002)	108.79(±0.0002)	109.22(±0.0002)	109.58(±0.0002)
0.100	108.39(±0.0003)	108.90(±0.0003)	109.35(±0.0003)	109.74(±0.0003)
0.200	108.57(±0.0002)	109.01(±0.0002)	109.49(±0.0002)	109.90(±0.0002)
0.300	108.74(±0.0002)	109.11(±0.0002)	109.61(±0.0002)	110.05(±0.0002)
0.400	108.90(±0.0002)	109.31(±0.0002)	109.73(±0.0002)	110.20(±0.0002)

	$S_V^* \times 10^6$ / (m^3·kg·mol^{-2})			
0.000	-7.31(±0.0042)	-7.31(±0.0042)	-7.33(±0.0042)	-7.36(±0.0042)
0.100	-7.24(±0.0048)	-7.25(±0.0047)	-7.27(±0.0048)	-7.31(±0.0048)
0.200	-7.17(±0.0042)	-7.20(±0.0041)	-7.22(±0.0041)	-7.27(±0.0042)
0.300	-7.11(±0.0040)	-7.14(±0.0040)	-7.17(±0.0040)	-7.22(±0.0041)
0.400	-7.04(±0.0041)	-7.08(±0.0041)	-7.12(±0.0042)	-7.17(±0.0042)

[a]m_B is the molality of aqueous solutions of 1-octyl-3-methylimidazolium bromide.

Table S4 Partial molar transfer volumes ΔV_ϕ^0 of L-threonine and Glycyl-L- threonine measured in 1-octyl-3-methylimidazolium bromide aqueous solutions at four temperatures (288.15, 298.15, 308.15, and 318.15 K) under constant pressure (p = 0.1 MPa).

[a]m_B / (mol·kg^{-1})	$\Delta V_\phi^0 \times 10^6$ / (m^3·mol^{-1})			
	T=288.15 K	T=298.15 K	T=308.15 K	T=318.15 K
	L-threonine			
0.100	0.30	0.33	0.35	0.38
0.200	0.60	0.66	0.71	0.76
0.300	0.89	0.98	1.14	1.33

0.400	1.17	1.29	1.52	1.49
		Glycyl-L-threonine		
0.100	0.19	0.11	0.14	0.16
0.200	0.36	0.22	0.27	0.32
0.300	0.53	0.32	0.40	0.47
0.400	0.69	0.52	0.52	0.62

[a] m_B is the molality of aqueous solutions of 1-octyl-3-methylimidazolium bromide.

Table S5 Measurements of sound velocity (u) and apparent molar isentropic compressibility ($K_{\phi,s}$) for L-threonine and Glycyl-L-threonine carried out in aqueous 1-octyl-3-methylimidazolium bromide solutions at 288.15–318.15 K and p=0.1 MPa pressure.

[a] m_A /(mol·kg^{-1})	u/ (m·s^{-1})				$K_{\phi,S}\times 10^{-6}$/(m^3.mol^{-1}.GPa^{-1})			
	T=288.15 K	T=298.15 K	T=308.15 K	T=318.15 K	T=288.15 K	T=298.15 K	T=308.15 K	T=318.15 K
				L-threonine + 0.000 mol·kg^{-1} [OMim] [Br]				
0.00000	1466.53[a]	1495.78[a]	1519.35[a]	1535.94[a]				
0.09947	1475.09	1503.34	1526.62	1543.31	-37.8033	-29.6141	-26.5900	-26.4704
0.11043	1476.03	1504.18	1527.42	1544.12	-37.7958	-29.6165	-26.5952	-26.4752
0.20746	1484.38	1511.56	1534.50	1551.30	-37.7274	-29.6357	-26.6389	-26.5157

0.30817	1493.05	1519.22	1541.86	1558.76	-37.6526	-29.6509	-26.6795	-26.5531
0.39894	1500.86	1526.12	1548.49	1565.48	-37.5821	-29.6607	-26.7121	-26.5828
0.49718	1509.31	1533.59	1555.67	1572.76	-37.5026	-29.6673	-26.7432	-26.6110
0.60278	1518.40	1541.62	1563.38	1580.58	-37.4137	-29.6700	-26.7721	-26.6368
0.69235	1526.11	1548.43	1569.93	1587.21	-37.3357	-29.6688	-26.7929	-26.6551
0.80693	1535.97	1557.15	1578.30	1595.69	-37.2324	-29.6628	-26.8150	-26.6740
0.98174	1551.01	1570.44	1591.07	1608.64	-37.0682	-29.6444	-26.8391	-26.6934
L-threonine + 0.100 mol·kg^{-1} [OMim] [Br]								
0.00000	1480.26[a]	1508.28[a]	1531.00[a]	1546.59[a]				
0.10861	1488.41	1516.43	1539.15	1554.74	-28.9309	-27.3042	-26.1892	-25.6273
0.19873	1495.16	1523.18	1545.90	1561.49	-28.9575	-27.3373	-26.2267	-25.6671
0.30237	1502.94	1530.96	1553.68	1569.27	-28.9833	-27.3707	-26.2652	-25.7082
0.41892	1511.68	1539.70	1562.42	1578.01	-29.0064	-27.4025	-26.3027	-25.7487
0.50745	1518.32	1546.34	1569.06	1584.65	-29.0199	-27.4227	-26.3273	-25.7755
0.61858	1526.65	1554.67	1577.39	1592.98	-29.0323	-27.4434	-26.3536	-25.8047
0.70929	1533.46	1561.48	1584.20	1599.79	-29.0387	-27.4566	-26.3714	-25.8249
0.79564	1539.93	1567.95	1590.67	1606.26	-29.0417	-27.4663	-26.3855	-25.8413
0.91483	1548.87	1576.89	1599.61	1615.20	-29.0413	-27.4750	-26.4003	-25.8593
0.99742	1555.07	1583.09	1605.81	1621.40	-29.0380	-27.4781	-26.4077	-25.8688
L-threonine + 0.200 mol·kg^{-1} [OMim] [Br]								
0.00000	1493.99[a]	1520.78[a]	1542.65[a]	1557.24[a]				

0.11042	1502.27	1529.06	1550.93	1565.52	-27.0927	-25.6986	-24.7405	-24.3079
0.21349	1510.00	1536.79	1558.66	1573.25	-27.1331	-25.7451	-24.7911	-24.3605
0.29738	1516.29	1543.08	1564.95	1579.54	-27.1622	-25.7794	-24.8287	-24.3997
0.39875	1523.90	1550.69	1572.56	1587.15	-27.1932	-25.8165	-24.8700	-24.4430
0.51217	1532.40	1559.19	1581.06	1595.65	-27.2226	-25.8528	-24.9110	-24.4863
0.59893	1538.91	1565.70	1587.57	1602.16	-27.2413	-25.8770	-24.9388	-24.5158
0.71081	1547.30	1574.09	1595.96	1610.55	-27.2611	-25.9037	-24.9702	-24.5495
0.80736	1554.54	1581.33	1603.20	1617.79	-27.2742	-25.9229	-24.9935	-24.5747
0.89872	1561.39	1588.18	1610.05	1624.64	-27.2834	-25.9378	-25.0124	-24.5955
0.98724	1568.03	1594.82	1616.69	1631.28	-27.2894	-25.9495	-25.0278	-24.6127
L-threonine + 0.300 mol·kg^{-1} [OMim] [Br]								
0.00000	1507.72[a]	1533.28[a]	1554.30[a]	1567.89[a]				
0.10847	1515.86	1541.42	1562.44	1576.03	-25.3526	-24.1692	-24.9636	-22.8783
0.19879	1522.63	1548.19	1569.21	1582.80	-25.3965	-24.2175	-25.0003	-22.9323
0.30523	1530.61	1556.17	1577.19	1590.78	-25.4434	-24.2696	-25.0389	-22.9911
0.39638	1537.45	1563.01	1584.03	1597.62	-25.4795	-24.3102	-25.0681	-23.0376
0.49891	1545.14	1570.70	1591.72	1605.31	-25.5158	-24.3517	-25.0969	-23.0856
0.61325	1553.71	1579.27	1600.29	1613.88	-25.5512	-24.3928	-25.1240	-23.1341
0.70566	1560.64	1586.20	1607.22	1620.81	-25.5759	-24.4223	-25.1423	-23.1696
0.79943	1567.68	1593.24	1614.26	1627.85	-25.5976	-24.4488	-25.1577	-23.2023
0.89184	1574.61	1600.17	1621.19	1634.78	-25.6158	-24.4718	-25.1697	-23.2313

0.99157	1582.09	1607.65	1628.67	1642.26	-25.6320	-24.4932	-25.1795	-23.2593
L-threonine + 0.400 mol·kg^{-1} [OMim] [Br]								
0.00000	1521.45[a]	1545.78[a]	1565.95[a]	1578.54[a]				
0.10215	1529.11	1553.44	1573.61	1586.20	-23.7036	-22.7108	-24.6134	-21.8177
0.21687	1537.72	1562.05	1582.22	1594.81	-23.7684	-22.7801	-24.6564	-21.8914
0.30721	1544.49	1568.82	1588.99	1601.58	-23.8152	-22.8305	-24.6862	-21.9452
0.41098	1552.27	1576.60	1596.77	1609.36	-23.8645	-22.8840	-24.7163	-22.0027
0.50765	1559.52	1583.85	1604.02	1616.61	-23.9063	-22.9297	-24.7404	-22.0522
0.61438	1567.53	1591.86	1612.03	1624.62	-23.9480	-22.9758	-24.7628	-22.1025
0.71479	1575.06	1599.39	1619.56	1632.15	-23.9831	-23.0151	-24.7800	-22.1457
0.81053	1582.24	1606.57	1626.74	1639.33	-24.0130	-23.0490	-24.7931	-22.1835
0.89732	1588.75	1613.08	1633.25	1645.84	-24.0372	-23.0768	-24.8023	-22.2148
0.99084	1595.76	1620.09	1640.26	1652.85	-24.0603	-23.1039	-24.8093	-22.2456
Glycyl-L-threonine +0.000 mol·kg^{-1} [OMim] [Br]								
0.00000	1466.53	1495.78	1519.35	1535.94				
0.01042	1467.74	1496.87	1520.37	1536.88	-55.0172	-44.7964	-38.6098	-33.0250
0.02015	1468.87	1497.90	1521.32	1537.75	-55.0064	-44.8007	-38.6219	-33.0441
0.02997	1470.01	1498.93	1522.29	1538.64	-54.9953	-44.8049	-38.6341	-33.0631
0.04069	1471.25	1500.05	1523.34	1539.60	-54.9830	-44.8093	-38.6471	-33.0838
0.04984	1472.31	1501.01	1524.23	1540.43	-54.9724	-44.8130	-38.6581	-33.1012
0.05981	1473.47	1502.06	1525.21	1541.32	-54.9607	-44.8168	-38.6699	-33.1201

0.07034	1474.69	1503.17	1526.24	1542.27	-54.9482	-44.8206	-38.6822	-33.1398
0.08079	1475.90	1504.26	1527.27	1543.21	-54.9357	-44.8243	-38.6943	-33.1593
0.08987	1476.95	1505.22	1528.16	1544.03	-54.9246	-44.8273	-38.7046	-33.1760
0.09843	1477.95	1506.12	1529.00	1544.80	-54.9141	-44.8300	-38.7142	-33.1916
Glycyl-L-threonine + 0.100 mol·kg^{-1} [OMim] [Br]								
0.00000	1480.26	1508.28	1531.00	1546.59				
0.01085	1481.51	1509.42	1532.06	1547.57	-51.4927	-42.6250	-36.8201	-31.5577
0.02037	1482.60	1510.42	1533.00	1548.42	-51.4859	-42.6308	-36.8331	-31.5770
0.03116	1483.84	1511.55	1534.05	1549.39	-51.4781	-42.6372	-36.8476	-31.5987
0.04025	1484.89	1512.51	1534.94	1550.21	-51.4713	-42.6424	-36.8596	-31.6169
0.04938	1485.94	1513.46	1535.84	1551.03	-51.4644	-42.6475	-36.8716	-31.6349
0.06013	1487.17	1514.59	1536.89	1552.00	-51.4561	-42.6533	-36.8856	-31.6560
0.06908	1488.20	1515.53	1537.77	1552.81	-51.4491	-42.6581	-36.8970	-31.6735
0.08143	1489.62	1516.83	1538.98	1553.92	-51.4393	-42.6644	-36.9126	-31.6973
0.09071	1490.69	1517.80	1539.89	1554.75	-51.4317	-42.6690	-36.9242	-31.7151
0.10726	1492.59	1519.54	1541.51	1556.24	-51.4180	-42.6769	-36.9445	-31.7464
Glycyl-L- threonine + 0.200 mol·kg^{-1} [OMim] [Br]								
0.00000	1493.99	1520.78	1542.65	1557.24				
0.01034	1495.18	1521.87	1543.65	1558.17	-48.7530	-40.5543	-34.5641	-30.1459
0.02061	1496.36	1522.94	1544.65	1559.09	-48.7483	-40.5620	-34.5800	-30.1674
0.02978	1497.41	1523.91	1545.54	1559.92	-48.7440	-40.5688	-34.5939	-30.1864

0.04034	1498.63	1525.02	1546.56	1560.87	-48.7389	-40.5764	-34.6099	-30.2082
0.04937	1499.67	1525.96	1547.44	1561.68	-48.7344	-40.5828	-34.6233	-30.2266
0.06039	1500.93	1527.12	1548.51	1562.68	-48.7287	-40.5905	-34.6396	-30.2490
0.07142	1502.20	1528.28	1549.58	1563.67	-48.7229	-40.5980	-34.6557	-30.2712
0.08072	1503.27	1529.26	1550.48	1564.50	-48.7178	-40.6041	-34.6691	-30.2897
0.08976	1504.31	1530.20	1551.36	1565.32	-48.7128	-40.6099	-34.6821	-30.3076
0.09971	1505.46	1531.25	1552.32	1566.21	-48.7071	-40.6162	-34.6961	-30.3272
Glycyl-L- threonine + 0.300 mol·kg^{-1} [OMim] [Br]								
0.00000	1507.72	1533.28	1554.30	1567.89				
0.01135	1509.03	1534.47	1555.41	1568.91	-46.1546	-38.5808	-33.4675	-28.7923
0.01989	1510.01	1535.37	1556.25	1569.68	-46.1527	-38.5884	-33.4808	-28.8107
0.02976	1511.14	1536.40	1557.22	1570.57	-46.1504	-38.5971	-33.4959	-28.8317
0.04015	1512.34	1537.50	1558.23	1571.50	-46.1478	-38.6060	-33.5118	-28.8538
0.05031	1513.51	1538.56	1559.23	1572.42	-46.1451	-38.6146	-33.5270	-28.8751
0.06173	1514.82	1539.76	1560.35	1573.45	-46.1419	-38.6241	-33.5440	-28.8989
0.07124	1515.91	1540.76	1561.28	1574.30	-46.1391	-38.6318	-33.5581	-28.9186
0.08019	1516.94	1541.70	1562.16	1575.11	-46.1363	-38.6390	-33.5711	-28.9370
0.09375	1518.50	1543.12	1563.49	1576.33	-46.1319	-38.6496	-33.5906	-28.9646
0.09921	1519.13	1543.70	1564.02	1576.82	-46.1301	-38.6538	-33.5984	-28.9757
Glycyl-L-threonin + 0.400 mol·kg^{-1} [OMim] [Br]								
0.00000	1521.45	1545.78	1565.95	1578.54				

0.01147	1522.77	1546.98	1567.07	1579.57	-43.6905	-36.6181	-31.8976	-27.4896
0.02058	1523.82	1547.94	1567.97	1580.39	-43.6905	-36.6275	-31.9125	-27.5096
0.02935	1524.83	1548.86	1568.83	1581.18	-43.6903	-36.6364	-31.9268	-27.5288
0.03981	1526.03	1549.96	1569.85	1582.12	-43.6899	-36.6468	-31.9436	-27.5515
0.05125	1527.34	1551.16	1570.97	1583.15	-43.6893	-36.6581	-31.9617	-27.5762
0.06084	1528.45	1552.17	1571.91	1584.02	-43.6887	-36.6673	-31.9768	-27.5967
0.06921	1529.41	1553.05	1572.73	1584.77	-43.6880	-36.6753	-31.9899	-27.6145
0.08037	1530.69	1554.22	1573.83	1585.77	-43.6870	-36.6858	-32.0071	-27.6380
0.09034	1531.84	1555.27	1574.80	1586.67	-43.6859	-36.6950	-32.0224	-27.6589
0.10275	1533.27	1556.57	1576.02	1587.79	-43.6844	-36.7062	-32.0411	-27.6847

[a]m_A is the molality of dipeptides in aqueous solutions of 1-octyl-3-methylimidazolium bromide. represents reference (53) from where the speed of sound values has been taken. Standard uncertainties *u* obtained are $u(u)$ = 0.05 m·s^{-1}; $u(m)$ = 2 × 10^{-5} mol·kg^{-1}; $u(T)$ = 0.001 K, and $u(p)$ = 0.01 MPa.

Table S6 Cartesian coordinates of DFT-optimised [OMIm][Br]–L-Threonine complex at B3LYP/6-311G(d,p) level of theory.

H	-0.80891	2.85909	-0.69533
C	-0.70018	3.28223	0.29184
N	-1.32436	2.82552	1.37139
C	-0.93257	3.62534	2.43210
C	-0.07964	4.54590	1.95568
N	0.05981	4.32357	0.59895
H	-1.27780	3.48818	3.44537
H	0.43113	5.33287	2.48919
C	-2.23667	1.69094	1.40748

H	-2.70732	1.57957	0.42486
H	-3.03573	1.89847	2.12880
C	-1.46993	0.43131	1.80009
H	-0.62309	0.30026	1.11896
H	-1.04541	0.55513	2.80372
C	-2.38003	-0.79718	1.77247
H	-3.21267	-0.63746	2.46890
H	-2.82326	-0.91134	0.77587
C	-1.67023	-2.09649	2.16295
H	-1.22458	-1.99030	3.15938
H	-2.42788	-2.88643	2.23786
C	-0.59629	-2.52557	1.15886
H	-1.01880	-2.53377	0.14704
H	0.22096	-1.79828	1.16793
C	-0.04688	-3.91469	1.49412
H	0.41293	-3.90197	2.48978
H	-0.87983	-4.62725	1.53946
C	0.96315	-4.42908	0.46587
H	0.51516	-4.42170	-0.53457
H	1.19586	-5.47454	0.70108
C	2.26313	-3.64042	0.44535
H	2.97293	-4.10420	-0.24714
H	2.10345	-2.61116	0.11443
H	2.72420	-3.61707	1.43774
Br	-0.16976	3.45949	-3.62349
C	0.89044	5.06259	-0.33678
H	1.55587	4.37493	-0.86400
H	1.50586	5.79047	0.20159
H	0.25415	5.60138	-1.04305
C	2.95593	-0.02853	-1.76230
H	3.52530	0.38496	-0.92234
H	3.23055	-1.08126	-1.87423

H	3.27738	0.51626	-2.65693
C	1.45810	0.15133	-1.52753
H	1.17813	-0.39103	-0.61830
C	0.60939	-0.35156	-2.71977
H	0.83652	0.25809	-3.60118
C	-0.89620	-0.19711	-2.44449
O	-1.64039	-0.65358	-3.48301
H	-2.54748	-0.34435	-3.28396
O	-1.44584	0.22288	-1.43744
N	0.88712	-1.77811	-3.05119
H	0.55118	-2.36375	-2.28765
H	0.28970	-2.00741	-3.85148
O	1.25616	1.54136	-1.24369
H	1.09727	2.01345	-2.09924

Table S7 Cartesian coordinates of DFT-optimised [OMIm][Br]–Glycyl-L-Threonine complex at B3LYP/6-311G(d,p) level of theory.

**Atom**	**X (Å)**	**Y (Å)**	**Z (Å)**
H	0.23745	-2.67646	-0.35479
C	0.98555	-3.31487	0.10101
N	2.20025	-2.90133	0.45912
C	2.86213	-3.94393	1.07130
C	2.01650	-5.00738	1.06657
N	0.85114	-4.58998	0.45587
H	3.86748	-3.83613	1.44689
H	2.13599	-6.01434	1.43394
C	2.66897	-1.51852	0.31191
H	2.35310	-1.17647	-0.67604
H	3.76037	-1.54248	0.33752
C	2.08390	-0.62136	1.39800

H	1.00145	-0.78986	1.42410
H	2.48580	-0.91586	2.37527
C	2.35594	0.85638	1.12398
H	3.43066	1.06823	1.20797
H	2.06642	1.08658	0.09022
C	1.56489	1.76490	2.06351
H	0.50092	1.51561	1.97230
H	1.83861	1.55250	3.10669
C	1.76991	3.25559	1.78299
H	2.81234	3.51083	2.01360
H	1.64111	3.44252	0.70767
C	0.81877	4.17147	2.57544
H	0.54957	3.68123	3.52196
H	1.34332	5.09490	2.84967
C	-0.46134	4.56513	1.82503
H	-0.18246	5.15662	0.94200
H	-1.05073	5.23180	2.46801
C	-1.33964	3.39499	1.37863
H	-2.27151	3.75819	0.93160
H	-0.83627	2.77828	0.62711
H	-1.60355	2.74981	2.22580
Br	-3.47328	-3.05031	-1.39930
C	-0.34539	-5.39832	0.24722
H	-0.10189	-6.24698	-0.39343
H	-1.10466	-4.77755	-0.23073
H	-0.71253	-5.75167	1.21188
N	-1.45415	-1.81689	1.43746
H	-1.97307	-2.49928	0.88405
H	-1.77107	-1.92820	2.39667
C	-1.82302	-0.48692	0.98319
H	-2.90688	-0.29562	0.97673
H	-1.37649	0.25580	1.65643

C	-1.27897	-0.19998	-0.40827
N	-1.89263	0.80629	-1.06356
H	-2.69812	1.25362	-0.65209
C	-1.51331	1.18849	-2.40958
H	-1.98866	2.15848	-2.59321
C	-0.00681	1.46342	-2.43602
O	-0.32730	-0.79904	-0.91461
C	-2.04940	0.19102	-3.47050
H	-1.42872	-0.71316	-3.44800
O	-3.39291	-0.13091	-3.16319
H	-3.38661	-0.84049	-2.49501
C	-2.04477	0.79727	-4.86623
H	-1.04169	1.10493	-5.16843
H	-2.70407	1.67251	-4.89119
H	-2.41885	0.06368	-5.58601
O	0.63970	0.86330	-3.43523
H	1.57540	1.12728	-3.38574
O	0.54313	2.20269	-1.64816
